# Bioinspired Fatty Acid Amide‐Based Slippery Oleogels for Shear‐Stable Lubrication

**DOI:** 10.1002/advs.202105528

**Published:** 2022-01-24

**Authors:** Jaehyeon Lee, Boram Kim, Ji Woong Lee, Chan Young Hong, Gwang Hoon Kim, Sang Joon Lee

**Affiliations:** ^1^ Department of Mechanical Engineering Pohang University of Science and Technology Pohang 37673 Korea; ^2^ Department of Chemistry Pohang University of Science and Technology Pohang 37673 Korea; ^3^ Department of Biological Sciences Kongju National University Gongju 32588 Korea

**Keywords:** anti‐biofouling, fatty acid amide, lubrication, oleogels

## Abstract

Liquid‐repellent technology is an efficient means of energy‐saving and biofouling avoidance. However, liquid‐repellent surfaces suffer from inefficient lubricant retention under shear flow and fouling problem in marine environment. Here, the authors demonstrate a fatty acid amide (FAA)‐based oleogel for stable and sustainable lubrication in marine environment. The lubrication management of marine creatures is emulated in synthetic oleogels by incorporating solid (FAA) and liquid lubricants into the molecular meshes of polymeric networks, with the nature‐derived solid lubricant providing multifunctional synergistic effects with liquid oil molecules for slippery property and remarkable anti‐biofouling. The lubricant‐confining gel achieves shear‐stable lubricity with efficient oil management. The oleogel provides continued lubrication without biofouling for approximately 4 months in marine field tests. The gel design provides a new paradigm for sustainable and shear‐stable lubrication in marine environment.

## Graphical abstract

A new shear‐stable lubrication strategy is proposed to be engineered at the molecular level, inspired by the lubricant management of marine creatures. The bioinspired lubricant confinement strategy enables efficient oil management through the synergetic effects of the solid and liquid lubricant molecules in the molecular meshes. The dual solid/liquid lubricant‐based gel system provides slippery properties with anti‐biofouling properties, enabling shear‐stable lubrication without biofouling for practical marine applications.

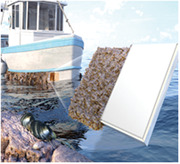

## 1 Introduction

Liquid‐repellent technologies have been a popular subject in both academic research and industrial applications over the past decades, including drag reduction, anti‐biofouling, self‐cleaning, anti‐icing, and medical applications.^[^ ^1^, ^2^, ^3^, ^4^, ^5^ ^]^ Inspired by the natural non‐wetting structures of lotus leaves and Nepenthes pitcher plants, synthetic liquid‐repellent surfaces have been developed as micro/nano‐structured surfaces on a composite solid–air interface and solid–liquid interface.^[^ ^6^, ^7^, ^8^ ^]^ Fundamental to these systems, micro/nano‐cavities of such surfaces are infused with non‐wetting lubricating materials such as air for superhydrophobic (SHP) surfaces and liquid lubricant for liquid‐infused surfaces (LISs).^[^ ^9^, ^10^ ^]^ Despite their promising liquid‐repellent and pressure‐stable properties, the lubricants trapped within the cavity cannot stand up to physical shear, so that the trapped lubricants, particularly those with low viscosity, can easily drain from the cavity under even low‐speed shear flow.^[^ ^11^, ^12^ ^]^ Moreover, flow‐induced shear at the flow/lubricant interface leads to an increase in surface roughness arising from curved lubricant meniscus and lubricant depletion, resulting in loss of frictional lubrication or even adverse effects on friction.^[^ ^13^ ^]^ Recent progress in extending the lubrication lifetime, by optimizing the rheological properties, patterned surface chemistry, surface geometry, and lubricant replenishment, remains an inherent limitation in lubrication under high‐speed turbulent flow, which severely restricts its practical applications such as in marine environments.^[^ ^11^, ^12^, ^14^, ^15^, ^16^, ^17^, ^18^, ^19^, ^20^, ^21^ ^]^ In addition, the fouling resistance of slippery surfaces enables to sustain their lubrication property without biofilm‐induced drag and corrosion in marine environments, indicating the requirement of multifunctionality in liquid‐repellent surfaces.

Different from previous microtexture‐based repellent technologies, marine creatures, such as coral, seaweed, and eel, exhibit slippery and anti‐biofouling properties utilizing a volumetric lubricant‐impregnated tissue system in which fatty acid‐incorporated tissues contain mucus.^[^ ^22^, ^23^, ^24^ ^]^ In addition, mussels have a waterproof outermost matrix based on fatty acid amide (FAA), primarily composed of oleamide, which is a commercial slip‐induced solid‐type lubricant.^[^ ^25^, ^26^ ^]^

**Figure 1:**
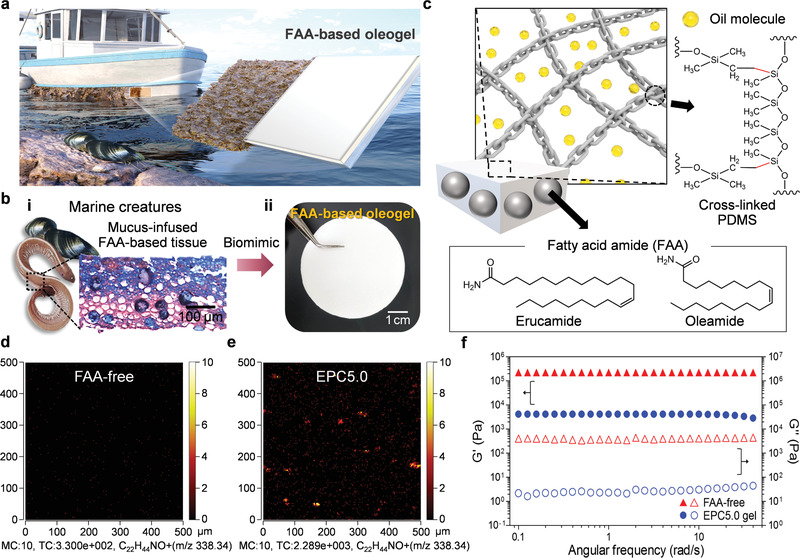
Design and characterization of FAA‐incorporating oleogels. a) Schematic illustration of FAA‐incorporating oleogels with shear‐stable lubrication in a marine environment. b) i) Histological cross‐section of skin tissue (blue, mucus; red, skin tissue of hagfish) of marine creatures showing mucus‐infused tissue, and ii) image of bioinspired FAA‐incorporating oleogel. c) Schematic structure of FAA‐incorporating oleogel with dual penetration of FAA and oil molecules in crosslinked PDMS polymeric network. ToF‐SIMS image showing the spatial distribution of erucamide (C_22_H_44_NO^+^, *m*/*z* 338.34) on a FAA‐free surface (d) and EPC5.0 surface (e). The color scale bar represents the range of pixel intensity [0, MC]. MC: maximum ion count per pixel, TC: total count in the entire image. f) Storage (G′, filled symbols) and loss moduli (G″, open symbols) of FAA‐free oleogel and EPC5.0 oleogel.

Inspired by this idea, we report a shear‐stable lubrication strategy based on FAA‐incorporating oleogels with dual solid/liquid lubricants to achieve sustainable lubrication in marine environment (**Figure** **1**a,b). We present a lubricant confinement strategy based on oil management through the synergetic effects of the solid and liquid lubricants in the molecular meshes of polymeric networks. The oil molecules are densely packed with high diffusive kinetics in the molecular mesh of the FAA‐incorporating oleogel. Gel‐confined solid/liquid lubricants form a molecularly continuous lubricant interface with little projected solid fraction and meniscus curvature. Furthermore, the FAA‐incorporating gel network eradicates lubricant loss under shear flow. Additionally, though most LISs rely on liquid lubricant to achieve high mobility, the dual solid/liquid lubricant system provides slippery properties with anti‐biofouling properties, indicating the multifunctional surfaces appropriate for various practical applications. In particular, the dual lubricant‐based gel system effectively resisted adhesion against various marine biological systems, compared to the conventional liquid lubricant‐based gel system. The epoch‐making multifunctional nature of FAA‐incorporating oleogels was stably sustained for approximately 4 months in long‐term marine field tests, indicating their strong potential for practical applications in marine environments.

## 2 Results

### 2.1 Fabrication and Characterization of FAA‐Incorporating Oleogels

A dual solid/liquid‐lubricant gel system was achieved by incorporating FAA as a solid slip agent and silicone oil molecules as a liquid slip agent into a polydimethylsiloxane (PDMS) polymeric network (Figure 1c). As migratory solid lubricants with high slip levels, unsaturated FAAs (erucamide and oleamide) were incorporated into PDMS to form diverse FAA‐incorporated composites (erucamide/PDMS composite (EPC) and oleamide/PDMS composite (OPC)) (Figure S1 and S2, Supporting Information). After the incorporation of FAAs into the polymer, the composite surfaces were morphologically flat and uniform to exhibit uniform surface property (Figure S3, Supporting Information). The chemical composition of EPC and OPC was confirmed through Fourier transform infrared (FTIR) and thermogravimetric analysis (TGA) (Figure S4 and S5, Supporting Information). The FAA‐incorporated composites were then organogelated through spontaneous diffusive penetration of silicone oil into the polymer matrix. The as‐prepared FAA‐incorporating oleogels were white, flexible, and elastic (Figure S6, Supporting Information).

The molecular content and spatial localization of erucamide on EPC surfaces were analyzed using time‐of‐flight secondary ion mass spectrometry (ToF‐SIMS). The EPC surface with erucamide content of 5.0 wt% was prepared (designated EPC5.0). The characteristic peak of erucamide on the EPC surfaces was identified by ToF‐SIMS spectra in positive mode (Figure S7, Supporting Information). Compared with FAA‐free surface, the characteristic erucamide ion image on the EPC5.0 surface exhibits a spatial distribution at *m*/*z* 338.34 (C_22_H_44_NO^+^) (Figure 1d,e). This FAA‐incorporating PDMS surface enables the formation of dual solid and liquid lubricant‐based gel surfaces when the PDMS portion is densely impregnated with oil molecules, which will be discussed later. Accordingly, FAAs on FAA‐incorporating surfaces can act as slip agents at the polymer surface. In addition, the incorporation of amphiphilic FAAs affects the surface chemistry with oil molecules in the gel state, which was confirmed through differences in the surface wettability of oleogels with different FAA types (Figure S8, Supporting Information).

For viscoelastic polymeric gels, additives penetrating the polymeric network can alter the material's ability to store and dissipate energy, which can be represented by the storage modulus (G′) and loss modulus (G″), respectively.^[^ ^27^ ^]^ In a wide linear viscoelastic region under oscillatory frequency sweep mode, the G′ values of both FAA‐free (PDMS gel) and FAA‐incorporating (EPC5.0 gel) oleogels are higher than the G″ values, confirming a crosslinked network (Figure 1f). The lower G′ values of EPC5.0 gel indicate fewer crosslinking points of the gel network than with the FAA‐free oleogel, while the lower G″ values of EPC5.0 gel are related to lower internal friction from restricted polymer chain motion (details in Figure S9, Supporting Information).^[^ ^28^ ^]^ These rheological results demonstrate the penetration of FAA chains into the PDMS polymeric network, indicating that the natural solid lubricant can be introduced into gel‐confined oil molecules.

### 2.2 Management of Oil Molecules in FAA‐Incorporating Oleogels

**Figure 2:**
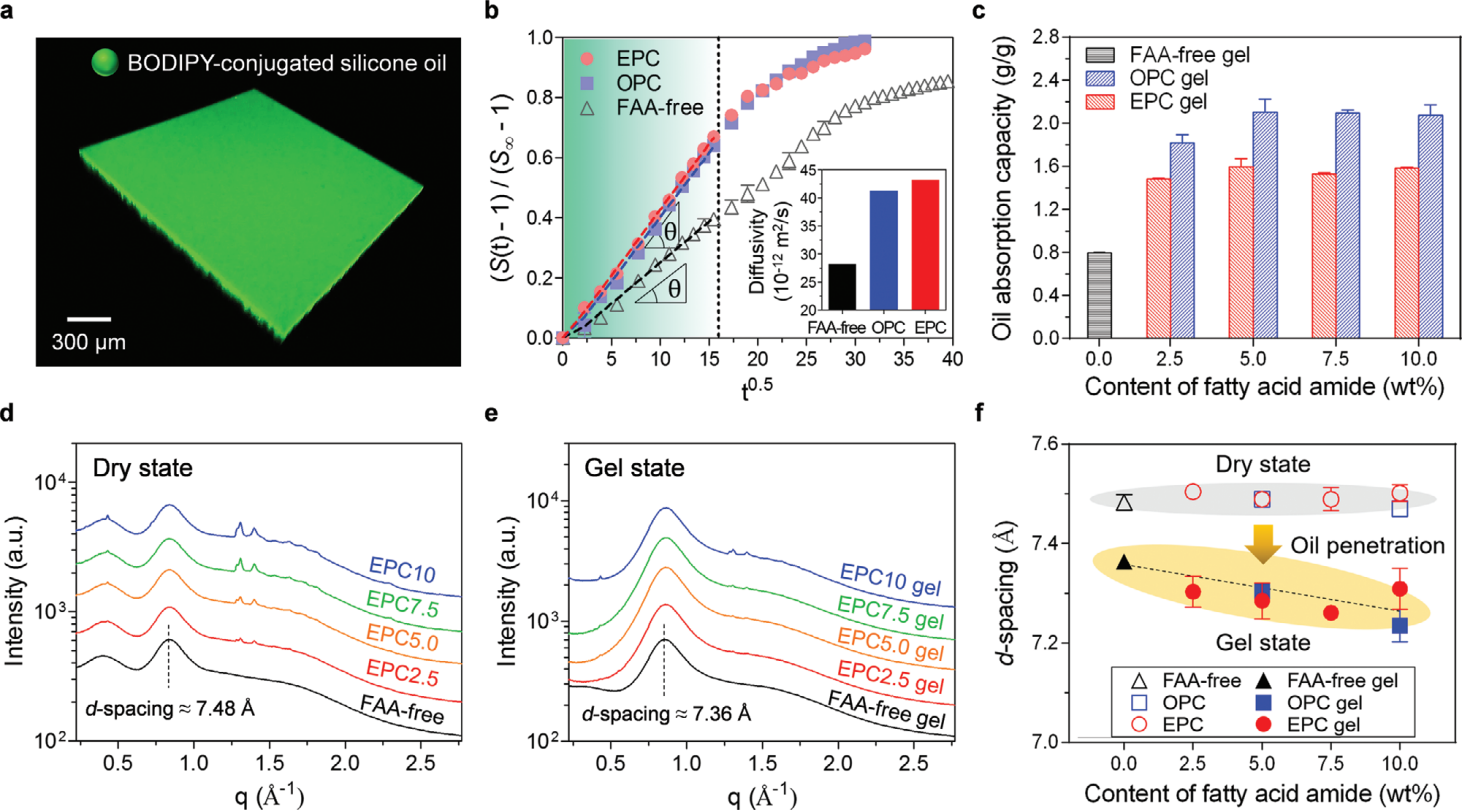
Tuneable oil transport and management of FAA‐incorporating oleogels. a) Confocal fluorescence image of EPC5.0 gel film using BODIPY‐conjugated silicone oil. b) Relative swelling ratio, (*S*(*t*)−1)/(*S*_∞_−1), of FAA‐free PDMS, OPC5.0, and EPC5.0 for silicone oil as a function of $\sqrt{t}$. Inset graph indicates the calculated oil diffusivity for each sample. c) Saturated oil content in FAA‐free and FAA‐incorporating oleogels per gram of corresponding gel samples. WAXS profiles for FAA‐free and FAA‐incorporating oleogel from dry (d) to gel (e) state. f) Spacing distance (*d*‐spacing) variations for FAA‐free and FAA incorporating oleogels from dry to gel state.

The high solvent‐compatibility of crosslinked PDMS in FAA‐incorporating oleogels enables diffusion of silicone oil into the polymeric network, which was confirmed by fluorescence imaging of the gel using BODIPY‐conjugated silicone oil (**Figure** **2**a). The diffusive absorption rates of silicone oil into the optimized thin EPC5.0 and OPC5.0 films (optimization in Figure S10, Supporting Information) were examined through their swelling dynamics to evaluate oil transport in FAA‐incorporating oleogels (Figure 2b). The swelling ratio of thin films in silicone oil (viscosity = 5cSt) was correlated with solvent diffusion through the relative swelling ratio (defined as (*S*(*t*)−1)/(*S*_∞_−1); *S*(*t*) and *S*_∞_ are transient/saturated swelling ratios in the length dimension, respectively) (details in Note S1, Supporting Information). Both EPC5.0 and OPC5.0 exhibit faster swelling behavior and reach their swollen steady state earlier than FAA‐free PDMS, indicating their fast oil absorption kinetics. The physical change in the swollen gels can be described by the mechanical properties of polymeric chains related to elasticity (Figure S10 and S12, Supporting Information).^[^ ^29^ ^]^ Accordingly, the diffusivities of oil molecules in EPC5.0 and OPC5.0, extracted from the curve slope *θ* in the transient swelling region (from the beginning to a black‐dotted vertical line in Figure 2b), are almost 1.5 times larger than that in PDMS (inset of Figure 2b, details in Note S1, Supporting Information). In addition, the saturated oil content of FAA‐incorporating oleogels is much higher than that of FAA‐free oleogels, representing increased oil absorption capacity (Figure 2c).

Oil management of FAA‐incorporating oleogels was further elucidated using wide‐angle X‐ray scattering (WAXS) analysis. In the WAXS profiles, a characteristic peak position of FAA‐free PDMS (at scattering vector (*q*) of ≈0.84 Å^−1^ in Figure 2d) shifts to a higher *q* value upon oil impregnation (FAA‐free oleogel in Figure 2e), indicating a decrease in the spatial correlation distance (d‐spacing = 2*π*/*q*) between polymeric chain segments. Given that the spacing distance decreases upon oil impregnation despite the swelling of the polymeric skeleton, the spacing distance of the oleogel is related to spatial correlations between penetrated silicone oil chains in the molecular matrix (details in Note S2, Supporting Information). From the WAXS profiles of FAA‐free and FAA‐incorporating oleogels (Figure 2e), the spatial correlations in the oleogel tend to decrease with increasing FAA content, indicating more densely packed oil molecules in the FAA‐incorporating gel network (Figure 2f). In other words, the hydrocarbon chains of amphiphilic unsaturated FAAs can act as oil‐wetting agents, which assist dense oil diffusion into the polymeric network and enable efficient management of gel‐confined oil molecules.^[^ ^30^ ^]^

### 2.3 Slippery Property and Shear‐Stability of FAA‐Incorporating Oleogel

**Figure 3:**
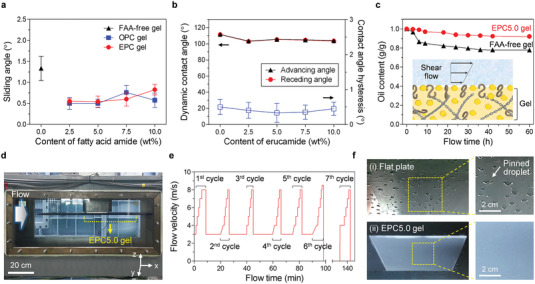
Shear‐stable water repellency of FAA‐incorporating oleogels. a) Sliding angle of 10 µL water droplet on FAA‐free and FAA‐incorporating oleogels. b) Dynamic water contact angle (advancing/receding angles, filled symbols, left y‐axis) and contact angle hysteresis (blue open symbols, right y‐axis) of EPC gels with different erucamide content. c) Temporal oil content in FAA‐free oleogel and EPC5.0 gel per gram of corresponding samples for boundary layer flow at *Re* of approximately 3.6 × 10^5^. Inset shows a schematic of shear‐stable lubrication from gel‐confined lubricant molecules. d) Experimental setup of a cavitation water tunnel to evaluate shear‐stability of EPC5.0 gel surface mounted on a submerged body with trailing edges. e) Repeated flow cycles with flow velocity up to 8 m s^−1^ exposed to the EPC5.0 gel. f) Optical images of a flat aluminum plate (i) and EPC5.0 gel‐coated plate (ii) after exposure to 7 flow cycles. After draining out water in the test section, water droplets pinned on the horizontally‐installed plates attached in the cavitation tunnel are observed from the bottom side direction of the test section.

FAA‐incorporating oleogels exhibit excellent water repellence, as signified by very low sliding angle (≈0.5° for 10 µl droplet, **Figure** **3**a) and very low contact angle hysteresis (≈0.4°, defined as the difference between advancing/receding contact angles, Figure 3b) for the optimized EPC5.0 gel and OPC5.0 gel. This high droplet mobility on F

AA‐incorporating oleogels demonstrates that water repellence can be amplified by integrating FAA and oil molecules into the hybrid configuration (Figures S13 and S14, Supporting Information). The enhanced slippery properties of FAA‐incorporating oleogels are less related to a result of dissolution and leaching of FAAs in silicone oil (Figure S15, Supporting Information). This highly slippery feature of FAA‐incorporating oleogels enables the slip flow over the surface (Figure S16, Supporting Information). In addition, FAA‐incorporating oleogels repel various liquids without surface contamination (Figure S17, Supporting Information).

To evaluate the sustainable slippery property of the FAA‐incorporating oleogels under shear flow, the oil retention capability of the EPC5.0 gel was tested in a circulating water tunnel. Compared to FAA‐free oleogel, the impregnated oil content of EPC5.0 gel was well retained with only 8% loss of oil mass for 60 h exposure to a shear flow at Reynolds number (Re), based on the distance from the leading edge, of about 3.6 × 10^5^ (Figure 3c).

Based on the efficient lubricant confinement strategy based on oil management (Inset of Figure 3c), FAA‐incorporating oleogel was exposed to turbulent flow in a cavitation water tunnel to demonstrate its shear‐stable lubrication durability (Figure S18, Supporting Information). After mounting the EPC5.0 gel‐coated plate (20 cm width × 20 cm length) on the submerged body with trailing edges inside the test section, water flowed at the freestream velocities of 3 to 8 m s^−1^ (Figure 3d). After exposure to a high‐speed flow for 7 cycles, the water in the tunnel was evacuated (Figure 3e). After 7 cycles, water droplets were pinned on a flat aluminum plate (Figure 3f(i)). However, even after exposure to a high‐speed flow for 7 cycles, the FAA‐incorporating oleogel exhibited high water repellence without droplet pinning, which confirms its shear‐stable lubrication (Figure 3f(ii)). In addition, after exposure to 1‐cycle high‐speed flow, the FAA‐incorporating oleogel retained their slippery property (Movie S1, Supporting Information).

### 2.4 Anti‐Biofouling Property for Sustainable Lubricity

**Figure 4:**
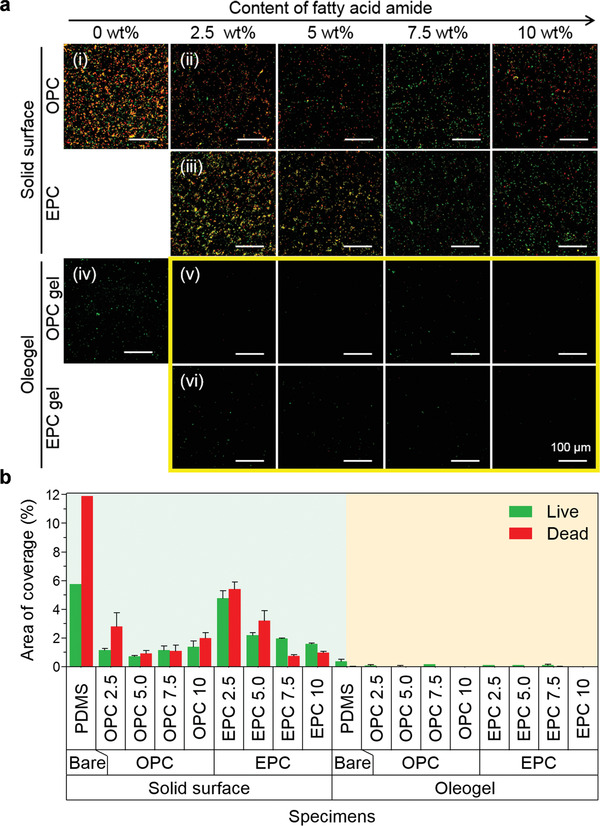
Anti‐biofouling property of FAA‐incorporating oleogels. a) Confocal microscopy images of *E. coli* cultured on i) a FAA‐free PDMS, ii) OPCs, iii) EPCs, iv) FAA‐free PDMS oleogel, v) OPC gels, vi) EPC gels with different FAA content for 24 h. The bacteria are stained with a fluorescent labeling agent (live/dead bacterial viability kit). Live cells are shown in green and dead cells are shown in red. The scale bar represents 100 µm. b) Quantification of coverage area of the live (green) and dead (red) cells cultured on solid samples (green region) and oleogel samples (orange region).

In a marine environment, the anti‐biofouling property of slippery surfaces on marine vessels is essential to sustain their slippery feature without biofilm‐induced drag and corrosion. To address these practical issues, slippery surfaces with marine antibiofouling property were experimentally investigated in consideration of marine applications.^[^ ^31^ ^]^ To assess the anti‐biofouling activity of FAA‐incorporating oleogels, *Escherichia coli* (*E. coli*) bacteria were cultured on various oleogels and control surfaces. After 24 h incubation, *E. coli* cultured on OPCs (**Figure** **4**a(ii)) and EPCs (Figure 4a(iii)) showed notably decreased adhesion compared to that grown on an FAA‐free PDMS surface (Figure 4a(i)). This intrinsic anti‐biofouling nature of FAAs is quantitatively confirmed through the coverage area of the attached bacteria (green region in Figure 4b).

After organogelation, the bacterial adhesion on FAA‐incorporating oleogels (Figure 4a (v,vi)) is significantly reduced compared to the case of the counterpart solid surfaces. In particular, EPC gels and OPC gels exhibit higher antifouling properties than FAA‐free oleogels, indicating a synergetic anti‐adhesive effect of FAA and oil molecules in the hybrid configuration. FAA‐incorporating oleogels with FAA contents exhibit almost zero attachment of bacteria on their surfaces. It is notable that *E. coli* on an FAA‐free oleogel (PDMS gel) were all alive, as evidenced by the green fluorescence emitted, indicating non‐toxicity of the impregnated silicone oil (details in Figure S19, Supporting Information). In addition, compared with FAA‐free oleogels, FAA‐incorporating oleogels also exhibited superior antibiofilm formation capability for marine bacteria (*M. dokdonensis*) and brown algae (*Cladosiphon sp*) (Figures S20 and S21, Supporting Information). In terms of surface energy, FAA‐incorporating oleogels have low surface energy in the range of 19.7–27.3 mJ m^−2^, which corresponds to the optimum surface energy range (20–30 mJ m^−2^) for minimum bioattachment in the Baier curve (see Figure S22, Supporting Information).^[^ ^32^ ^]^ In addition, the highly anti‐adhesive nature of FPC oleogels is related to the low pull‐off force required to separate foulants from the surface, which mitigates foulant adhesion (details in Figure S23, Supporting Information).^[^ ^33^ ^]^ These results imply that the dual solid/liquid lubricant gel system provides a multifunctional surface with shear‐stable lubrication and antifouling appropriate for marine applications.

### 2.5 Long‐Term Shear‐Stable Lubrication in Marine Field Tests

**Figure 5:**
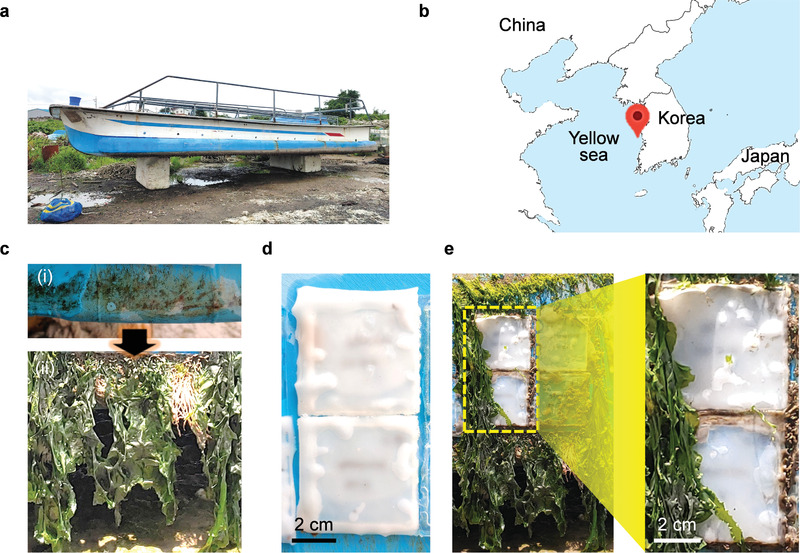
Long‐term marine field test attached to an operating ship. a) Photograph of a ship model. b) Local map showing the location (red marker) of marine field test at the Yellow Sea. c) Optical image of a ship surface before the test (i) and after approximately 4 months (ii). Photograph of EPC5.0 gel on the ship before the test (d) and after approximately 4 months (e).

To demonstrate the long‐term lubrication properties under harsh marine environments with shear flow, optimized EPC5.0 gel‐coated plates (see optimization in Figures S24–S26, Supporting Information) were attached to a commercial ship (**Figure** **5**a). Here, three EPC5.0 gel‐coated surfaces were attached to the ship (Figure 5d). The underwater distance of the attached samples was approximately 0.3 m from sea level, indicating a hydrostatic pressure of 1.043 bar on the gel. The ship was operated for approximately 4 months (from 13 Feb 2020 to 8 June 2020) in the Yellow Sea at the latitude of 36°08”12.5“N and longitude of 126°32”27.4”E near the Korean city of Seocheon (Figure 5b). On average, the ship operated at a velocity of 27.8 km h^−1^ (from 18.5 to 55.6 km h^−1^) for 6 h per day. After operation, the ship floated on the shore, indicating that the gel surfaces were continuously exposed to marine environments. Seawater in this marine field test contained diverse marine organisms, indicating harsh fouling conditions (Figure S26, Supporting Information). Even after 1‐month field test, seaweeds deposited on FAA‐free (PDMS), OPC, and EPC surfaces attached to the ship, indicating the necessity of a dual lubricant gel system (Figure S27, Supporting Information). During the 4‐month field test, the ship surfaces were covered with various marine organisms such as seaweeds, which can induce an increase in hydrodynamic volume and additional friction (Figure 5c). However, EPC5.0 gel‐coated surfaces exhibit non‐biofilm formation on the surfaces for 4 months under shear flow conditions, which strongly demonstrates their long‐term anti‐adhesive and shear‐stable lubrication properties (Figure 5e). Furthermore, FAA‐incorporating oleogels exhibit diverse substrate compatibility (Figure S28, Supporting Information) and high scalability (Figure S29, Supporting Information), indicating strong potential for large‐scale practical applications in the future.

## 3 Conclusion

Designing liquid‐repellent surfaces from FAA‐incorporating oleogels allows shear‐stable lubrication to be engineered at the molecular level for practical applications under various flow conditions. Inspired by non‐wetting slippery marine creatures, the lubrication attributes inherent to amphiphilic FAAs are translated to the properties of oleogel networks with synergistic effects of oil molecules. The shear‐stable slippery surface is created by incorporating solid and liquid lubricant into the molecular meshes of the gel, which provides high lubricant retention under shear flow. This unique gel design enabled persistent lubrication under shear flow conditions. A dual lubricant‐based gel system generates multifunctional properties, including sustainable slippery property and anti‐biofouling properties. As demonstrated in marine field tests using a ship, FAA‐incorporating oleogels were able to sustain their multifunctional properties in an almost perfect manner under marine environments for approximately 4 months. Thus, FAA‐based oleogel design can pave the way for a shear‐stable multifunctional liquid‐repellent surface for many practical applications, including drag reduction, anti‐biofouling, anti‐icing, and self‐cleaning technology.

## 4 Experimental Section

### Chemicals and Materials

Erucamide and oleamide were purchased from Tokyo Chemical Industry (Japan). Polydimethylsiloxane (PDMS, Sylgard 184) was purchased from Dow Chemical (MI, USA). Silicone oil and toluene were purchased from Sigma Aldrich (USA). Primer coating (1200 OS primer) was purchased from Dow Corning (USA) to improve adhesion of cured silicones to various substrates.

### Preparation of FAA‐Incorporating Composites

A mixture of polydimethylsiloxane (PDMS) prepolymer and curing agent was prepared at a weight ratio of 10:1. For the 10 g of the PDMS mixture solution, erucamide powders were prepared at different weight percentages ranged from 2.5 to 10 wt%. For example, 0.25641 g of the erucamide powder was prepared for 10 g of the PDMS solution to fabricate EPC with 2.5 wt% erucamide content. The erucamide powder was completely dissolved in toluene with a volume of 4 mL under an ultrasonic sonication at 70 °C for 2 h. Then, the erucamide‐toluene solution was quickly added to 10 g of the prepared PDMS solution and mixed using a vortex generator for 3 h. The mixture was then coated onto a flat plate using a doctor blading method (micrometer adjustable film applicator, MTI). The coated film was vacuumed and cured at 61 °C in a vacuum oven for 25 h to remove the toluene used for the prepared mixture.

For the fabrication of OPC surfaces, the oleamide replaced the erucamide and whole fabrication procedure was conducted the same.

### Preparation of FAA‐Free PDMS Surface (Control Sample)

A mixture of polydimethylsiloxane (PDMS) prepolymer and curing agent was prepared at a weight ratio of 10:1. The mixture was coated onto a flat plate using a doctor blading method. The coated film was cured at 61 °C in a vacuum oven for 24 h.

### Preparation of FAA‐incorporating Oleogels

A primer solution was spin‐coated on a flat plate and dried in ambient condition for at least 20 min. The prepared erucamide‐PDMS mixture in S.1.2.1. was then coated onto the primer‐coated flat plate using a doctor blading method. For the preparation of EPC gel itself without coating on the substrates, the primer was not used and the erucamide‐PDMS mixture was coated on the bare flat plate. The coated film was vacuumed and cured at 61 °C in a vacuum oven for 25 h to remove the toluene used for the prepared mixture. The cured EPC film was immersed in silicone oil overnight to fabricate the EPC gel surface.

For the fabrication of the OPC gel surface, the oleamide replaced the erucamide and whole fabrication procedure was conducted the same.

### Preparation of FAA‐Free Oleogels (Control Sample)

A primer solution was spin‐coated on a flat plate and dried in ambient condition for at least 20 min. The prepared PDMS solution (mixture of PDMS prepolymer and curing agent at a weight ratio of 10:1) was then coated onto a primer‐coated flat plate using a doctor blading method. For the preparation of the FAA‐free PDMS gel itself without coating on the substrate, the primer was not used and the PDMS mixture was coated on the bare flat plate. The coated film was vacuumed and cured at 61 °C in a vacuum oven for 25 h. The cured PDMS‐coated surface was immersed in silicone oil overnight to fabricate the FAA‐free PDMS gel surface.

### Material Characterization

The wettability property of test surfaces was investigated by measuring their water contact angles. After dripping sessile deionized water droplets with a volume of 5 µL on the surfaces, static contact angles were measured using a SmartDrop instrument (Femtofab, Korea). The sliding angles of water droplets with a volume of 10 µl were measured for each surface. The thickness of test samples was measured using a profilometer (Alpha‐Step D‐500, Tencor Instruments) with an accuracy of 0.1 µm. To investigate the oil absorption capacity, the temporal weight changes of the oil‐absorbing oleogels were analyzed using an electronic mass balance (AP250D, Ohaus, USA) with an accuracy of 0.01 mg. The FTIR spectrometer was conducted using a PerkinElmer instrument. The TGA was conducted using a TA instrument (Q50). The sample was heated with a heating rate of 10 °C min^−1^ to 800 °C under nitrogen atmosphere. Tensile mechanical properties of test samples were measured using a microforce testing machine (Flexible Materials Tester, Hansung Systems, Inc.) with a 50 N load cell. Samples were stretched with an extension rate of 3 mm min^−1^ at 23 °C.

## Conflict of Interest

The authors declare no conflict of interest.

## Supporting information

Supporting Information

Supplemental Movie 1

## Data Availability

The data that support the findings of this study are available from the corresponding author upon reasonable request.
