# Supplementary material for: Bioinspired Fatty Acid Amide‐Based Slippery Oleogels for Shear‐Stable Lubrication
Source: Adv Sci (Weinh). 2022 Jan 24;9(8):2105528. doi: 10.1002/advs.202105528 (PMC8922109; doi:10.1002/advs.202105528)
Supplement: Supplementary file 1 — Supporting Information [file ADVS-9-2105528-s001.pdf]

ADVANCED SCIENCE
Open Access

Supporting Information

for *Adv. Sci.*, DOI: 10.1002/advs.202105528

Bioinspired Fatty Acid Amide-based Slippery Oleogels for Shear-stable Lubrication

*Jaehyeon Lee, Boram Kim, Jiwoong Lee, Jiwoong Lee, Chan Young Hong, Kwanghoon Kim, Sang Joon Lee**

Supplementary Information

Bioinspired fatty acid amide-based slippery oleogels for shear-stable lubrication

Jaehyeon Lee, Boram Kim, Jiwoong Lee, Jiwoong Lee, Chan Young Hong, Kwanghoon Kim, Sang Joon Lee*

*Correspondence to: sjlee@postech.ac.kr

Table of content

1. Supplementary Methods

2. Supplementary Notes (Note. S1 to S2)

3. Supplementary Figures (Fig. S1 to S29)

4. Reference List

Supplementary Information

Table of content

S1. Supplementary Methods

S1.1 Chemicals and materials

S1.2. Fabrication procedure

S1.2.1. Preparation of FAA-incorporating composites

S1.2.2. Preparation of FAA-free PDMS surfaces (control sample)

S1.2.3. Preparation of FAA-incorporating oleogels

S1.2.4. Preparation of FAA-free oleogels (control sample)

S1.3. Material characterization

S1.4. Histological staining of marine creature

S1.5. Rheological measurements

S1.6. Visualization of impregnated oil in FAA-incorporating oleogels

S1.7. Wide-Angle X-ray Scattering (WAXS) experiments

S1.8. Oil retention capability test under shear flow

S1.9. Velocity fields of flow over FAA-incorporating oleogels

S1.10. Shear-stable lubrication in cyclic high-speed flow test

S1.11. Anti-biofouling property test

S1.11.1. Antibacteria bioassay experiment

S1.11.2. Anti-marine bacteria bioassay experiment

S1.11.3. Anti-brown algae bioassay experiment

S1.12. Long-term marine field test

S1.12.1. Marine field test in a Yellow Sea farm

S1.12.2. Marine field test attached to an operating ship

S2. Supplementary Notes

S2.1. Diffusive transport of oil molecules in FAA-incorporating oleogels

S2.2. Effect of FAAs on oil management in FAA-incorporating oleogels

S3. Supplementary Figures

S3.1. Preparation of FAA-incorporating composites
S3.2. NMR spectra of FAA-incorporating PDMS composites
S3.3. Surface morphology of FAA-incorporating composite surfaces
S3.4. Chemical composition of FAA-incorporating composites
S3.5. Flexibility and elasticity of FAA-incorporating oleogels
S3.6. Molecular content analysis of FAA-incorporating composite surfaces
S3.7. Surface wettability of FAA-incorporating oleogels
S3.8. Dual penentration of fatty acid amide and oil molecules in PDMS network
S3.9. Swelling ratio of the wall structure in FAA-incorporating oleogels
S3.10. Mechanical property of FAA-incorporating oleogels
S3.11. Elasticity of FAA-incorporating oleogels
S3.12. Water repellence of FAA-incorporating oleogels
S3.13. Solubility of FAA for silicone oil
S3.14. Slip flow over FAA-incorporating oleogel surfaces
S3.15. Liquid repellence of FAA-incorporating oleogels
S3.16. Experimental setup of a high-speed water cavitation tunnel test
S3.17. Anti-bacteria property of FAA-incorporating oleogels
S3.18. Anti-marine bacteria property of FAA-incorporating oleogels
S3.19. Anti-brown algae biofilm assay experiment
S3.20. Optimum surface energy of FAA-incorporating oleogels for anti-bioadhesion
S3.21. Low pull-off force of FAA-incorporating oleogels
S3.22. Long-term marine field test near sea farm
S3.23. Long-term marine field test attached to an operating ship
S3.24. Diverse substrate compatibility of FAA-incorporating oleogels
S3.25. Scalability of FAA-incorporating oleogels

S1. Supplementary Methods

S1.1. Chemicals and materials

Erucamide and oleamide were purchased from Tokyo Chemical Industry (Japan). Polydimethylsiloxane (PDMS, Sylgard 184) was purchased from Dow Chemical (MI, USA). Silicone oil and toluene were purchased from Sigma Aldrich (USA). Primer coating (1200 OS primer) was purchased from Dow Corning (USA).

S1.2. Fabrication procedure

S1.2.1. Preparation of FAA-incorporating composites

A mixture of polydimethylsiloxane (PDMS) prepolymer and curing agent was prepared at a weight ratio of 10:1. For the 10 g of the PDMS mixture solution, erucamide powders were prepared at different weight percentages ranged from 2.5 to 10 wt%. For example, 0.25641 g of the erucamide powder was prepared for 10 g of the PDMS solution to fabricate EPC with 2.5 wt% erucamide content. The erucamide powder was completely dissolved in toluene with a volume of 4 mL under an ultrasonic sonication at 70 °C for 2 h. Then, the erucamide-toluene solution was quickly added to 10 g of the prepared PDMS solution and mixed using a vortex generator for 3 h. The mixture was then coated onto a flat plate using a doctor blading method (micrometer adjustable film applicator, MTI). The coated film was vacuumed and cured at 61 °C in a vacuum oven for 25 h to remove the toluene used for the prepared mixture.

For the fabrication of OPC surfaces, the oleamide replaced the erucamide and whole fabrication procedure was conducted the same.

S1.2.2. Preparation of FAA-free PDMS surfaces (control sample)

A mixture of polydimethylsiloxane (PDMS) prepolymer and curing agent was prepared at a weight ratio of 10:1. The mixture was coated onto a flat plate using a doctor blading method.

The coated film was cured at 61 °C in a vacuum oven for 24 h.

S1.2.3. Preparation of FAA-incorporating oleogels

A primer solution was spin-coated on a flat plate and dried in ambient condition for at least 20 min. The prepared erucamide-PDMS mixture in S.1.2.1. was then coated onto the primer-coated flat plate using a doctor blading method. For the preparation of EPC gel itself without coating on the substrates, the primer was not used and the erucamide-PDMS mixture was coated on the bare flat plate. The coated film was vacuumed and cured at 61 °C in a vacuum oven for 25 h to remove the toluene used for the prepared mixture. The cured EPC film was immersed in silicone oil overnight to fabricate the EPC gel surface.

For the fabrication of the OPC gel surface, the oleamide replaced the erucamide and whole fabrication procedure was conducted the same.

S1.2.4. Preparation of FAA-free oleogels (control sample)

A primer solution was spin-coated on a flat plate and dried in ambient condition for at least 20 min. The prepared PDMS solution (mixture of PDMS prepolymer and curing agent at a weight ratio of 10:1) was then coated onto a primer-coated flat plate using a doctor blading method. For the preparation of the FAA-free PDMS gel itself without coating on the substrate, the primer was not used and the PDMS mixture was coated on the bare flat plate. The coated film was vacuumed and cured at 61 °C in a vacuum oven for 25 h. The cured PDMS-coated surface was immersed in silicone oil overnight to fabricate the FAA-free PDMS gel surface.

S1.3. Material characterization

The wettability property of test surfaces was investigated by measuring their water contact angles. After dripping sessile deionized water droplets with a volume of 5 μL on the surfaces,

static contact angles were measured using a SmartDrop instrument (Femtofab, Korea). The sliding angles of water droplets with a volume of 10 μl were measured for each surface. The thickness of test samples was measured using a profilometer (Alpha-Step D-500, Tencor Instruments) with an accuracy of 0.1 μm. To investigate the oil absorption capacity, the temporal weight changes of the oil-absorbing oleogels were analysed using an electronic mass balance (AP250D, Ohaus, USA) with an accuracy of 0.01 mg. The Fourier transform infrared (FTIR) spectrometer were conducted using a PerkinElmer instrument. The thermogravimetric analysis (TGA) was conducted using a TA instrument (Q50). The sample was heated with a heating rate of 10 °C/min to 800 °C under nitrogen atmosphere. Tensile mechanical properties of test samples were measured using a microforce testing machine (Flexible Materials Tester, Hansung Systems, Inc.) with a 50 N load cell. Samples were stretched with an extension rate of 3 mm/min at 23 °C. The surface morphology was examined using an atomic force microscopy (AFM, VECO Dimension 3100). The captured AFM images were reconstructed using Nanoscope V (version 7.0) software to obtain 3D structural information and quantitative surface roughness. Nuclear magnetic resonance (NMR) spectra was obtained using ^{1}H NMR (Bruker 500) spectroscopy at 300 Hz using $CDC1_3$.

S1.4. Histological staining of a marine creature

Skin tissue of Hagfish (Eptatretus stoutii) was stained with Alcian blue for mucus and safranin O for tissue to observe mucus in skin tissue. Biosample was fixed in 10% formalin solution for 24 h at 4°C. After fixation, the sample was embedded in paraffin. The sample was cut to 8 μm in thickness using a rotary microtome (Finess ME, Thermo). The sliced sample was deparaffined and stained with 1% w/v Alcian blue (pH 2.5) and 0.1% w/v Safranin O solution. The stained sample was observed using a microscope (Zeiss Axiovert 200) with a 10× objective lens.

S1.5. Rheological measurements

Rheological properties were measured using a rheometer (DHR-2, TA Instruments). Parallel plate geometry with a diameter of 20 mm was used. Oscillation frequency sweep mode was performed to measure the rheological properties of test samples in the range of the linear viscoelasticity. The angular frequency range was between 0.1 and 100 Hz at a fixed strain of 0.1% and temperature of 25°C.

S1.6. Visualization of impregnated oil in FAA-incorporating oleogels

To visualize the impregnated silicone oil in the FAA-incorporating oleogel, the silicone oil with a viscosity of 5 cSt was stained with 4,4-difluoro-1,3,5,7,8-pentamethyl-4-bora-3a, 4a-diaza-s-indacene (BODIPY 493/503, Invitrogen Corporation). The BODIPY dye was used in this study due to its nonpolar structure and previous usage as an oil tracer.[1] The BODIPY dye was dissolved in dimethylsulfoxide (DMSO) at a concentration of 2.5 mg/mL. 10 mL of silicone oil was dissolved in 20 mL of dichloromethane (DCM). The silicone oil-DCM solution was mixed with 400 μL of the BODIPY-DMSO solution for 1 h. The DMSO and DCM in the mixed solution were removed using a rotary evaporator.

After immersing the EPC5.0 surface into the prepared BODIPY-stained oil, the BODIPY-stained oil impregnated in the surface was examined with a confocal microscope (TCS SP5II MP, Leica Microsystems) with a 40× objective lens (Leica Microsystems). The obtained confocal images were analysed using LAS AF 2.7 software (Leica Microsystems).

S1.7. Wide-Angle X-ray Scattering (WAXS) experiments

Synchrotron WAXS measurements were performed at the 4C Beamline of the Pohang

Accelerator Laboratory (PAL, Pohang, Korea). Film samples were prepared and mounted on the stage. The sample-to-detector distance was 20 cm to cover a high scattering vector q range from 0.2 to 2.8 Å^{-1} ($q = 4\pi \sin(\theta/2)/\lambda$, where θ is the scattering angle and λ is the wavelength of the incident X-ray beam equal to 0.733 Å). Two-dimensional scattering patterns were azimuthally averaged to obtain one dimensional scattering intensity profiles versus q.

S1.8. Oil retention capability test under shear flow

Oil retention performance of a FAA-incorporating oleogel surface was measured for a boundary layer flow in a circulating water channel with a test section of 1.15 m in length, 0.3 m in width, and 0.03 m in height. The test samples were fixed on the bottom of the test section. The samples were located 1 m downstream from the entry point of the test section. The Reynolds numbers (Re_L) from the entry point was about 3.6×10^5. The oil mass change of each sample was measured using an electronic mass balance with an accuracy of 0.01 mg.

S1.9. Velocity fields of flow over FAA-incorporating oleogel surfaces

FAA-incorporating oleogel-coated plate was positioned on one side of a rectangular acrylic channel (length: 250 mm, width: 24 mm, height (H): 10 mm). Salt water with NaCl was used as the working fluid and PMMA-Rhodamine-B particles with a mean diameter of 20–50 μm were seeded into the working fluid as tracer particles. Considering the density of fluorescent tracing particles, NaCl was dissolved in deionized water at a concentration of 0.3457 g/ml to satisfy the neutral buoyancy of the tracer particles in the working fluid.

Particle image velocimetry (PIV) and particle tracking velocimetry (PTV) techniques were conducted together to measure the velocity field information of the flow in the region near

oleogel surfaces. Flow images were captured by a high-speed complementary metal oxide semiconductor (CMOS) camera (FASTCAM Mini UX100, Photron, Japan) with 1280 × 1024 pixels at 50 frames per second (fps). A thin laser sheet was generated by a 100mW continuous diode-pumped solid-state laser (Crystal Laser, USA) with a wavelength of 532 nm and optical lenses. The laser sheet was aligned with the centerline of the channel. The working fluid in the channel was driven by a syringe pump (PHD 2000, Harvard Apparatus, USA). The Reynolds number (Re) was 40. Three hundred instantaneous velocity fields were consecutively captured for each experimental condition.

In the region near the test sample (y/H = 0 to 0.1) and acrylic channel surface (y/H = 0.9 to 1), near-wall velocity profiles were measured by adopting a two-frame PTV technique through a peak-intensity-searching algorithm to determine displacements of individual particles3. In the center region of the channel (y/H = 0.1 to 0.9), the captured images were processed with PIV technique (PIVview 2C, PIVTEC, Germany). A multigrid interrogation window was operated with a fast Fourier transform-based cross-correlation PIV algorithm to extract instantaneous velocity fields with an interrogation window size of 64 × 64 pixels with 50% overlapping. The whole velocity profile over the samples in the channel was then obtained by combining the velocity field information obtained by the PIV and PTV techniques. The measured instantaneous velocity fields were ensemble-averaged to obtain the mean velocity field, and the slip length was evaluated.

S1.10. Shear-stable lubrication in cyclic high-speed flow test

The shear-stable lubrication of the FAA-incorporating oleogel surface for a high-speed turbulent boundary layer flow was measured in a cavitation water tunnel at the Korea Research Institute of Ships and Ocean Engineering (KRISO, Daejeon, Korea). The test section of the cavitation channel was 2.6 m in length, 0.6 m in width, and 0.6 m in height.

The EPC5.0 gel was coated with a thickness of 100 μm on a flat rectangular aluminium plate with 20 cm × 20 cm. The EPC5.0 gel-coated plates were mounted on a flat plate model (950 mm in length, 340 mm in width, and 2 mm in thickness) with a leading edge and trailing edge. The AL6061 aluminium plate was used as the flat plate to minimize the distortion. The aluminium plate was anodized to prevent corrosion in the water during the cyclic repeated tests. The flatness of the anodized aluminium plate was approximately 0.001 mm. The flat plate model mounted with EPC5.0 gel surfaces was connected with a surrounding strut and then fixed on the top of the test section. The test plate was aligned to make it parallel to the flow direction in the cavitation tunnel. After mounting the plate in the test section, water speed increased from 3 to 8 m/s at an interval of 1 m/s in one experiment. The experiment was cyclically repeated 7 times. Water temperature was maintained at about 9 °C during the experiment.

S1.11. Anti-biofouling property test

S1.11.1. Antibacteria bioassay experiment

To evaluate the antibacterial properties of FAA-incorporating oleogel samples, gram-negative *Escherichia coli* (*E.coli*, ATCC25404) bacteria were purchased from American Type Culture Collection (ATCC, Manassa, VA). In the bioassay analysis, a stock solution of *E.coli* was streaked onto TYE (tryptone and yeast extract) dehydrated agar plates for the growth of bacterial colony. The plates were placed in an incubator at 37 °C overnight, and then stored at 4 °C. A single colony was picked from the streak plates and inoculated into 5 mL of sterile Luria broth (LB Broth Miller, BD Difco, USA) to prepare the *E.coli* broth. The *E.coli* was grown for 24 h with shaking at 200 rpm at 37 °C. After incubation, the *E.coli* was diluted to 1/5 in the sterile Luria broth.

FAA-incorporating oleogel-coated glass cover slips (sized 18 × 18 mm, Duran group, Germany) were wiped with an oil paper to remove external oil layer on the surface and placed in 6-well plates. FAA-free PDMS, EPCs, OPCs, and FAA-free PDMS gel surfaces were tested as control groups. Test samples were incubated with 3 ml of the bacterial suspension for 24 h at 37 °C. After incubation, test samples were rinsed with phosphate-buffered saline (PBS) solution to remove the non-adherent bacteria and then fixed with 10% formalin solution. The fixed samples were rinsed with PBS solution and deionized water.

The test samples were then stained with a fluorescent labeling reagent (live/dead bacterial viability kit, BacLight™, L7012, Molecular Probes, Invitrogen, Grand Island, NY). The stained samples were incubated for 15 min in the dark at room temperature and then rinsed twice with PBS. The samples were then examined with a confocal microscope (TCS SP5II MP, Leica Microsystems) with a 40× (zoom ×3) objective lens (Leica Microsystems). Field of view was 123.02 × 123.02 μm. The obtained confocal images were analysed using LAS AF 2.7 software (Leica Microsystems). The areal coverage of the bacteria was quantified using Image J software (NIH, Bethesda, MD, USA).

S1.11.2. Anti-marine bacteria bioassay experiment

Maribacter dokdonensis (*M. dokdonensis*) was isolated from the seawater of Jindo, Korea. M. dokdonensis was cultured on a marine agar (Marine Agar 2216, BD Difco, USA) plate at 21 ºC for 7 days and then the plate was stored at 4 °C. A single colony was picked from streak plates and inoculated into 5 mL sterile marine broth (Marine Broth 2216, BD Difco, USA) to prepare M. dokdonensis broth. *M. dokdonensis* was then grown for 24 h with shaking at 200 rpm at 21 °C. After incubation, the *M. dokdonensis* solution was diluted to 1/5 into the sterile marine broth.

FAA-incorporating oleogel-coated glass cover slips (18 × 18 mm, Duran group, Germany)

were wiped with an oil paper to remove external oil layer on the surface and placed in 6-well plates. Bare glass, FAA-free PDMS, EPCs, OPCs, and FAA-free PDMS gel surfaces were tested as control groups. The test samples were incubated with 3 mL of *M. dokdonensis* solution for 24 h at 21 °C incubator. After incubation, the samples were rinsed with PBS solution to remove the non-adherent bacteria.

The test samples were then stained with a fluorescent labeling reagent (live/dead bacterial viability kit, BacLight, L7012, Molecular Probes, Invitrogen, Grand Island, NY). The stained samples were incubated for 15 min in the dark at room temperature and then rinsed twice with PBS solution. The samples were then examined with a confocal microscope (TCS SP5II MP, Leica Microsystems) with a 40× (zoom ×3) objective lens (Leica Microsystems). Field of view was 123.02 × 123.02 μm. The acquired confocal images were analysed using LAS AF 2.7 software (Leica Microsystems).

S1.11.3. Anti-brown algae bioassay experiment

Haploid gametophyte brown algae (*Cladosiphon sp*) were used to evaluate the biofouling properties of FAA-incorporating oleogel surfaces. Brown algae were incubated in integrated microbiome resource (IMR) medium at 20 °C under the illumination of 20 μmol photons m^{-2} s^{-1} (12 h light:12 h dark).[2] The gametes were finely chopped, and the upper solution was removed using centrifugation. The chopped brown algae were washed three times and diluted to 1 mg/mL with the IMR medium.

FAA-incorporating oleogel-coated glass cover slips (18 × 18 mm, Duran group, Germany) were wiped with an oil paper to remove external oil layer on the surface and placed in 6-well plates. Bare cover glass, FAA-free PDMS, EPCs, OPCs, FAA-free PDMS gel surfaces were tested as control groups. The test samples were cultured with 5 mL of the diluted algae suspension for 48 h at 20 °C under 20 μmol photons m^{-2} s^{-1}. The medium was replaced to

wash the non-adhesive ones at 20 °C under 20 μmol photons m^{-2} s^{-1}. The incubation and observation procedures were performed continuously for 3 weeks.

The samples were then observed using a stereomicroscope (Olympus SZX10, Olympus, Tokyo, Japan) attached to an internet protocol camera (IP 8000; Hanwha Techwin, Changwon, Korea). Field of view was 5.5 × 4.3 mm. The areal coverage of the algae was quantified using Image J software (NIH, Bethesda, MD, USA).

S1.12. Long-term marine field test

S1.12.1. Marine field test in a Yellow Sea farm

The EPC gels and OPC gels with different FAA contents (2.5 wt%, 5.0 wt%, 7.5 wt%, 10 wt%) were coated on flat aluminium plates with the size of 10 × 3.3 cm^2. The bare acrylic, FAA-free PDMS, EPCs with different erucamide contents (2.5 wt%, 5.0 wt%, 7.5 wt%, 10 wt%), OPCs with different oleamide contents (2.5 wt%, 5.0 wt%, 7.5 wt%, 10 wt%), FAA-free PDMS gel surfaces (sized 5 × 3.3 cm^2) were prepared as control groups.

The long-term marine field test in a marine farm was conducted in the Yellow Sea located at the latitude of 36°08'12.5"N and longitude of 126°32'27.4"E near the Korean city of Seocheon. The test place was located next to a seaweed farm (kelp farm), where marine organisms and seaweeds are easily attached to general surfaces. The test samples were installed in a frame. In the frame installation, the films coated on the substrates were exposed to the seawater and the substrates were faced the frame wall. The frame was immersed in seawater at a depth of 1.5 m from sea level. In other words, a hydrostatic pressure of 1.1604 bar was applied to the test samples during the long-term field test. The salinity and temperature of seawater were ranged from 31.5 to 33.0 ppt and from 6.8 to 24.3 °C during the test, respectively. The pH of seawater was 8.1 ± 0.1.

The deposition of marine organisms and seaweeds on the test samples were examined using a digital camera. The marine organisms adhered on acrylic panels were observed using a stereo microscope (Olympus SXZ1). In addition, the marine organisms were further observed using a microscope (BX50, Olympus, Tokyo, Japan) attached to an camera (DP72, Olympus, Tokyo, Japan) with the aid of cellSens software (Olympus, Tokyo, Japan). The field test was conducted continuously for 11 weeks (from 25 March 2020 to 8 June 2020).

S1.12.2. Marine field test attached to an operating ship

The long-term marine field test using a ship was conducted in the Yellow Sea located at the latitude of 36°08'12.5"N and longitude of 126°32'27.4"E near the Korean city of Seocheon. The test samples were attached to a commercial FRP ship (fiber reinforced plastic ship, 8.1 m in length, 3.63 m in width, 0.88 m in height, 3.56 ton in weight). When the ship was floated on the sea, the underwater distance of the attached samples was approximately 0.3 m from sea level. In other words, a hydrostatic pressure of 1.0427 bar was applied to the test samples during the long-term field test. The salinity, temperature, and pH conditions of seawater were the same as the long-term marine farm test.

The ship operated 6 h daily on average. During the daily operation, the minimum and maximum cruising speed of the ship was approximately 18.5 km/h and 55.6 km/h, respectively. The average cruising speed was 27.8 km/h. After the daily operation, the ship floated on the shore. The marine field test was conducted continuously for approximately 4 months (from 13 Feb 2020 to 8 June 2020).

S2. Supplementary Notes

Supplementary Note S1

Diffusive transport of oil molecules in FAA-incorporating oleogels

Oil transport in polymeric gels is linked to their swollen behavior. Oil molecules are diffused into the molecular matrix of FAA-incorporating composites, because polymer chains extend to maximize the interaction between a compatible solvent and cross-linked PDMS polymer network.[3] The temporal swelling behavior of FAA-incorporating composite films in a silicone oil bath was measured to characterize the diffusive transport of oil molecules into the molecular matrix of the FAA-incorporating composites. In this experiment, the FAA-free PDMS film with ca. 0.021 mm thickness, EPC5.0 with ca. 0.017 mm thickness, and OPC 5.0 with ca. 0.017 mm thickness were immersed in silicone oil with a viscosity of 5 cSt. The swollen geometry of a thin polymer film in swelling solvent was previously correlated with solvent diffusion as follows[7]:

$$\frac{S(t)-1}{S_\infty-1}=\frac{L(t)/L_0-1}{L_\infty/L_0-1}=\frac{2}{d}\sqrt{\frac{D}{\pi}}\times\sqrt{t} \qquad (1)$$

where L_0 is the initial length of the film, $L(t)$ is the length of the swollen film at swollen time t, L_∞ is the saturated length of the swollen film, $S(t)$ is the transient swelling ratio ($=L(t)/L_0$), S_∞ is the saturated swelling ratio ($=L_\infty/L_0$), d is the film thickness, and D is the diffusivity. Thus, the relative swelling ratio, ($S(t)$-1)/(S_∞-1), of FAA-free PDMS, EPC5.0, and OPC5.0 films were plotted as a function of $\sqrt{t}$. The relative swelling ratio presents a linear dependence on $\sqrt{t}$ in the initial transient swelling region. In this region, the slope (θ) of the swelling curve was obtained by linear curve fitting. Thus, the diffusivity of oil molecules in each polymer film was extracted from the slope as follows:

$$D=\pi(\frac{d}{2}\theta)^2 \qquad (2)$$

Supplementary Note S2

Effect of FAAs on oil management in FAA-incorporating oleogels

Effect of FAAs on the oil management in FAA-incorporating oleogels was deeply elucidated using synchrotron wide-angle X-ray scattering (WAXS) experiments. WAXS technique hs been utilized for conformational analysis of flexible polymers like PDMS.[5] The spatial correlation between polymeric chain segments was analysed by obtaining the spacing distance (*d*-spacing) as follows:

$$d\text{–}spacing = \frac{2\pi}{q_{max}} \quad (3)$$

where *d-spacing* is the spatial correlation distance between chain segments, and $q_{\max}$ is the scattering vector (q) at the maximum scattering intensity (I_{max}) of the broad peak.

Firstly, the initial spacing distances of dry-state polymeric chains were investigated for FAA-free (PDMS) and FAA-incorporating (EPC and OPC) films without impregnation of silicone oil. WAXS profiles of the FAA-free PDMS displayed a broad maximum PDMS peak at $q_{\max}$ value of ca. 0.84 Å^{-1} (Fig. 2d in the manuscript), which is commonly observed in X-ray diffraction (XRD) and small-angle X-ray scattering (SAXS) at high q-range ($q \approx$ 0.84~0.87 Å^{-1}).[6] The position ($q_{\max}$) of the PDMS peak slightly shifted to lower q values in the FAA incorporation (erucamide for EPCs and oleamide for OPCs), indicating a decrease in the equivalent spacing distance (see *d-spacing* plot in Fig. 2f of the manuscript).

After impregnation of silicone oil (PDMS liquid, with viscosity of 5 cSt) into each solid film, silicone oil molecules are penetrated into each molecular matrix with a swelling process. The positions ($q_{\max}$) of the PDMS peak shifted to higher q values from dry state to gel state, indicating a decrease in the spacing correlation distance between polymeric chain segments. After oil penetration into the PDMS, the polymeric skeleton of the PDMS swells as shown in Fig. 2b of manuscript, which induces an increase in interplanar spacing of PDMS

chains. However, the spacing distance for FAA-free (PDMS gel) and FAA-incorporating (EPC gels and OPC gels) oleogels decreased because of the spatial correlations between the penetrated oil chains in molecular matrix (comparison of *d-spacing* in Fig. 2f of the manuscript) of the oleogel. In particular, the spatial correlation distances for the FAA-incorporating oleogels was smaller than for the FAA-free oleogel and linearly decreased with increasing the FAA content in the gel. Accordingly, when FAA chains are incorporated into the PDMS network, oil molecules become more densely packed in the crosslinking gel network.

S3.1. Preparation of FAA-incorporating composites

Fatty acid amide(FAA)/PDMS composites were prepared as erucamide/PDMS composite (EPC) and oleamide/PDMS composite (OPC) depending on the type of FAAs (erucamide and oleamide). The EPCs with the erucamide content of 2.5, 5.0, 7.5, and 10 wt% were prepared and denoted as EPC2.5, EPC5.0, EPC7.5, and EPC10, respectively (Fig. S1(a)). The OPCs with the oleamide content of 2.5, 5.0, 7.5, and 10 wt% were prepared and denoted as OPC2.5, OPC5.0, OPC7.5, and OPC10, respectively (Fig. S1(b)). The content of FAAs is defined as follows:

$$\text{Weight content (wt\%)} = \frac{\text{Weight of fatty acid amide}}{(\text{Weight of PDMS}) + (\text{Weight of fatty acid amide})} \times 100$$

For example, 0.25641 g of erucamide powder was prepared for 10 g of PDMS mixture solution (prepolymer and curing agent at weight ratio of 10:1) to fabricate EPC2.5.

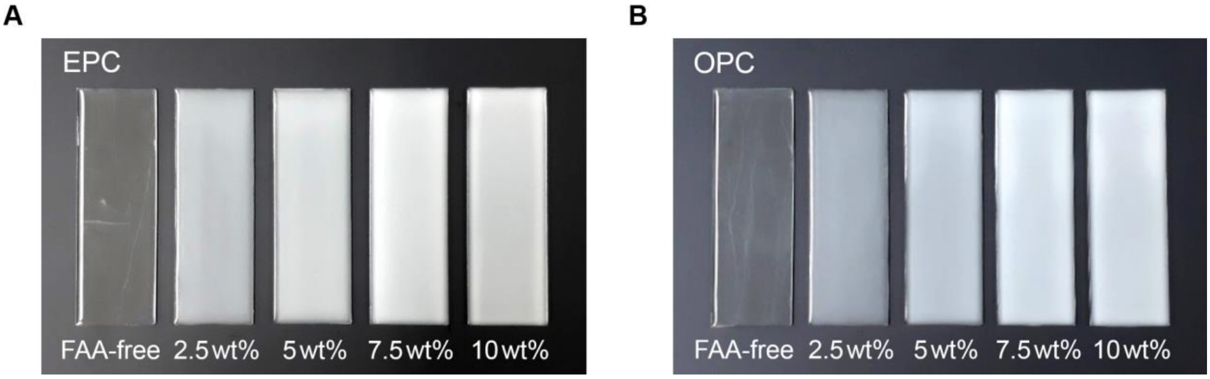

Figure S1. Optical images of (a) FAA-free (PDMS) and erucamide/PDMS composite (EPC) surfaces with different erucamide content, and (b) FAA-free PDMS and oleamide/PDMS composite (OPC) surfaces with different oleamide content.

S3.2. NMR spectra of FAA-incorporating PDMS composites

The unsaturated FAAs were incorporated into PDMS to form diverse FAA-incorporated composites. The chemical structure of the FAA/PDMS composites was investigated by ^{1}H NMR spectrum. In the ^{1}H NMR spectrum of the oleamide, the C=C bond (marked as ‘f’) of the oleamide exhibited a peak at 5.37 ppm (Fig. S2). The FAA-incorporating PDMS composites (OPC2.5, OPC5.0, and OPC7.5) also showed the C=C bond peaks with almost same integration. In other words, when FAAs were incorporated into PDMS, the C=C bonds of the oleamide in the FAA-incorporating PDMS composites was not reacted with the hydrogens of silyl-terminated PDMS via hydrosilylation. Accordingly, the incorporation of FAAs into PDMS results in the FAA-incorporating PDMS composites.

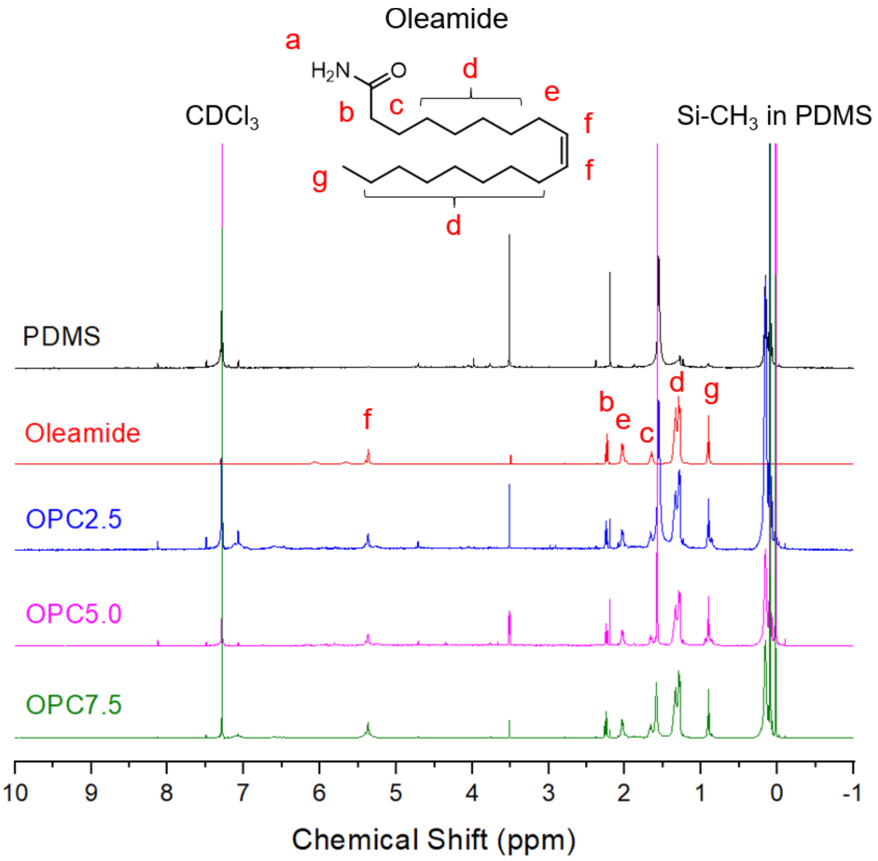

Figure S2. ^{1}H nuclear magnetic resonance (NMR) spectra of a crosslinked PDMS, oleamide, and FAA-incorporating composites with different oleamide content (OPC2.5, OPC5.0, and OPC7.5) in $CDCl_3$. Characteristic peaks of oleamide are marked in the spectrum.

S3.3. Surface morphology of FAA-incorporating composite surfaces

The surface roughness of a solid can affect its surface energy that is correlated with the contact angle of a liquid. In addition, the surface roughness can affect the friction coefficient of the liquid-repellent surfaces. The surface morphologies of the FAA-free (PDMS) and FAA-incorporating composite (EPCs and OPCs) surfaces were observed through AFM topography (Fig. S3). The FAA-free and FAA-incorporating surfaces exhibited flat surfaces with nanoscopic roughness. In other words, the flatten surface of the FAA-incorporating surfaces could represent the morphological and spatial uniformity of the incorporated solid lubricants in the composites.

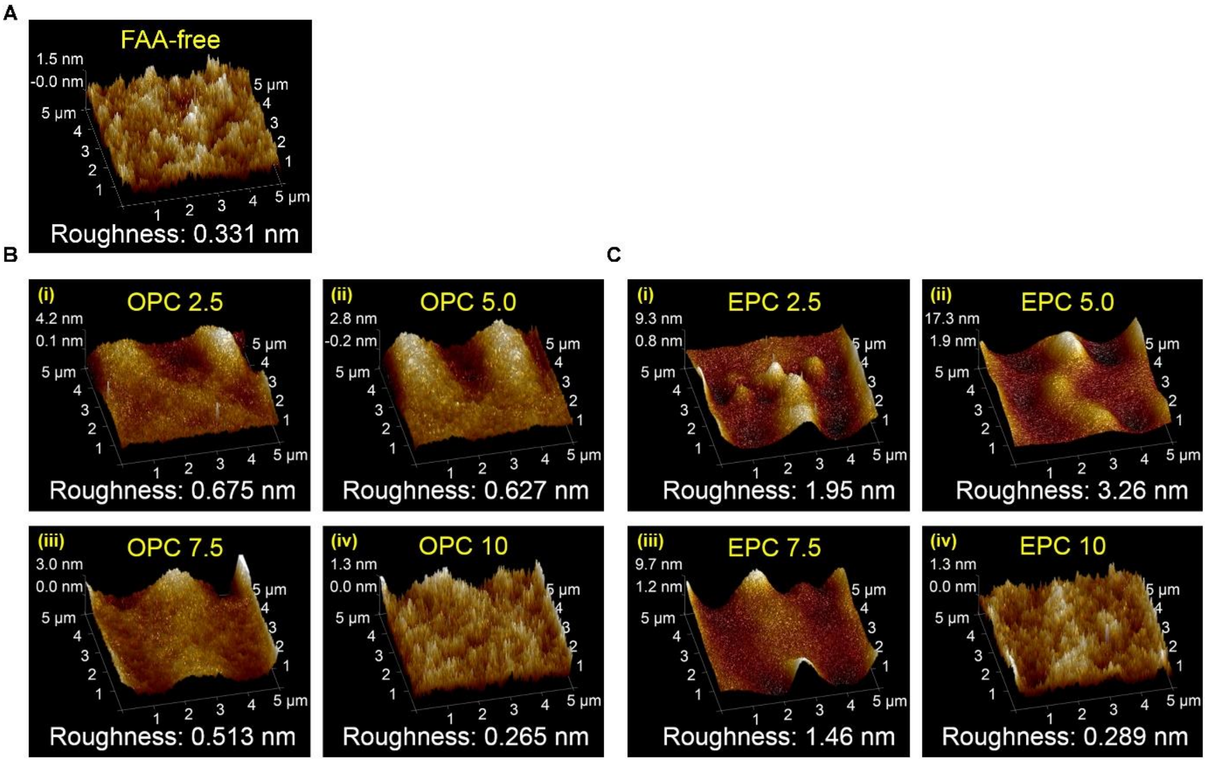

Figure S3. AFM topographic images of (a) FAA-free (PDMS), (b) OPCs with different oleamide content ((i) 2.5, (ii) 5.0, (iii) 7.5, (iv) 10 wt%), and (c) EPCs with different erucamide content ((i) 2.5, (ii) 5.0, (iii) 7.5, (iv) 10 wt%). The average surface roughness of each surface is written with each image. The scan area was 5.0 × 5.0 μm^2.

S3.4. Chemical composition of FAA-incorporating composites

The chemical composition of the FAA-incorporating composites was analysed by attenuated total reflection Fourier transform infrared (ATR-FTIR) spectroscopy. The spectrum of the FAA-free PDMS showed characteristic peaks (indicated by black arrows in Fig. S4) located at 1014 and 1070 cm^{-1} (Si-O-Si symmetric and asymmetric stretching, respectively), 1410 cm^{-1} (asymmetric bending of CH_3 groups), and 2906 cm^{-1} (symmetric stretching of CH_3 groups).[7] All the characteristic peaks of the PDMS were found in the FTIR spectra of the FAA-incorporating composites (EPC5.0 and OPC5.0). In the spectra of the FAA-incorporating composites (EPC5.0 and OPC5.0), new characteristic peaks (indicated by purple arrows in Fig. S2) appeared at 1632 cm^{-1} (NH_2 bending of the amide group), 1659 cm^{-1} (C=O stretching of the amide group), 2852 and 2923 cm^{-1} (CH_2 symmetric and asymmetric stretching, respectively), and 3186 and 3359 cm^{-1} (symmetric and asymmetric stretching of the NH_2 group, respectively).[8] These characteristic peaks of the FAA-incorporating composites confirm the presence of erucamide and oleamide in the PDMS network.

These results were confirmed with the thermogravimetric analysis (TGA). In the TGA curve of the OPC5.0 (blue curve in Fig. S5(a)), the weight loss started from 200 to 260 °C for the oleamide and continued from 300 °C for the PDMS. Those weight losses indicates the disintegration of the oleamide and PDMS polymer chains. Accordingly, the weight loss of the OPC could be attributed to rapid decomposition of the oleamide (from 200 °C to 260 °C) and gradual decomposition of the PDMS (from 300 °C to 800 °C). In addition, the initial weight loss of the OPC5.0 (from 200 to 260 °C) was ca. 5 %, which confirms the oleamide content (5 wt%) of the OPC5.0.

In the TGA curve of the EPC5.0 (blue curve in Fig. S5(b)), the weight loss started from 240 to 300 °C for the erucamide and continued from 300 °C for the PDMS, indicating the disintegration of the erucamide and PDMS polymer chains. Accordingly, the weight loss of

the EPC could be attributed to rapid decomposition of the erucamide (from 240 °C to 300 °C) and gradual decomposition of the PDMS (from 300 °C to 800 °C). In addition, the initial weight loss of the EPC5.0 (from 240 to 300 °C) was ca. 5 %, which confirms the erucamide content (5 wt%) of the EPC5.0.

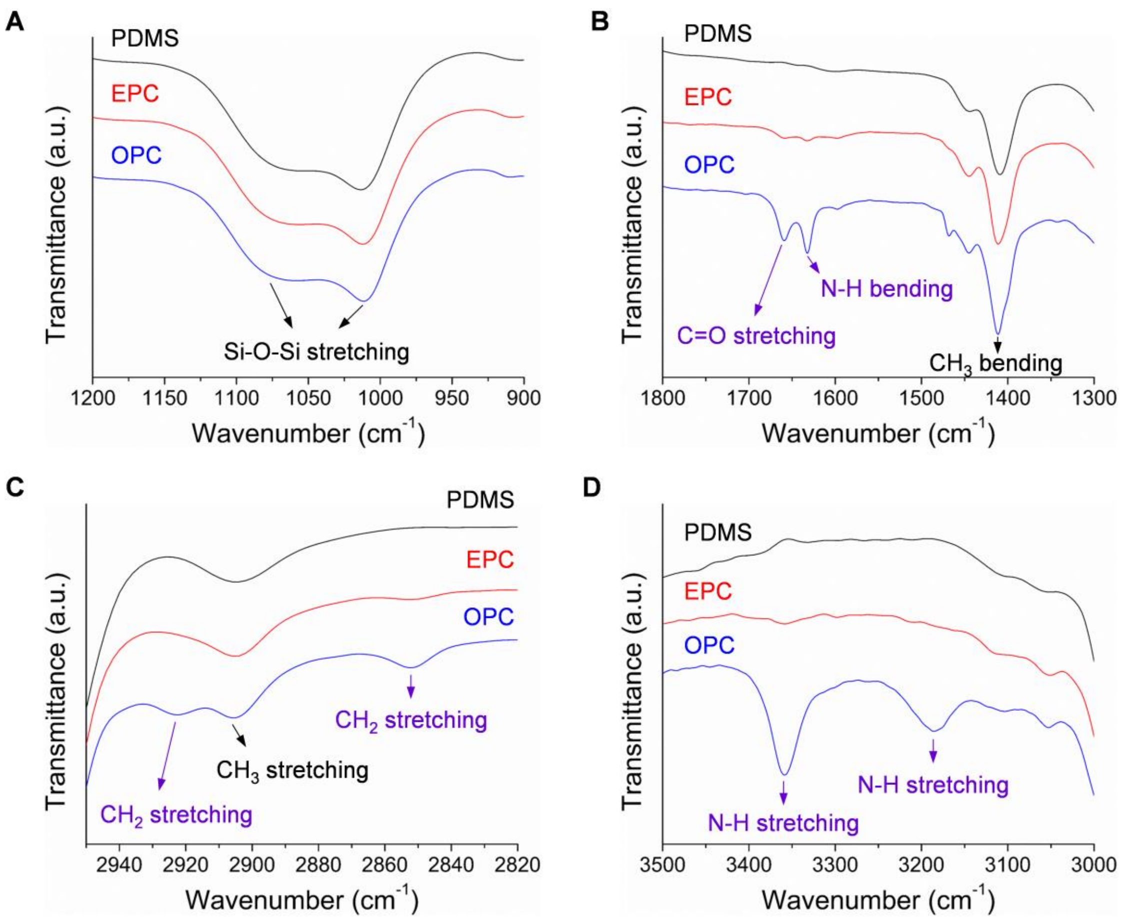

Figure S4. FTIR spectra of the FAA-free PDMS (black curve), EPC5.0 (red curve), and OPC5.0 (blue curve); the characteristic peaks of the PDMS (black arrows) and FAAs (purple arrows) are assigned within.

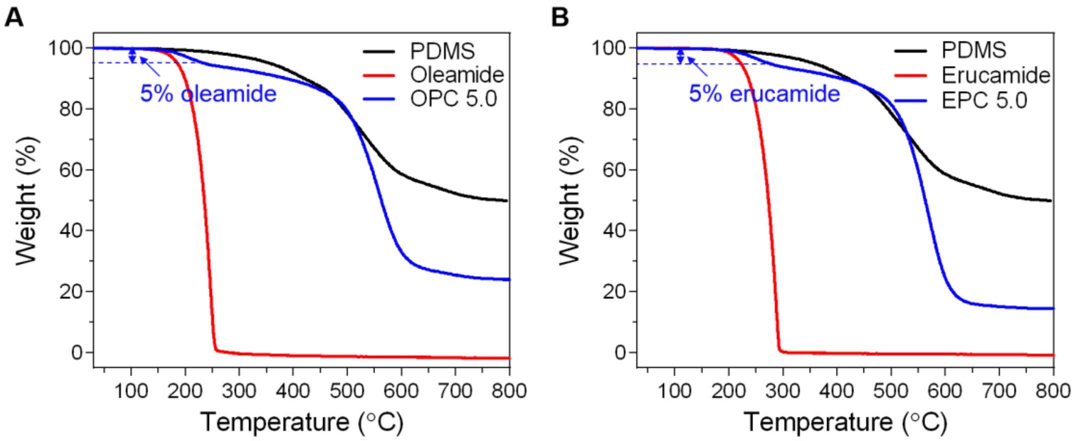

Figure S5. (a) TGA curves of the cured PDMS, oleamide, and OPC5.0. (b) TGA curves of the cured PDMS, erucamide, and EPC5.0.

S3.5. Flexibility and elasticity of FAA-incorporating oleogels

The FAA-incorporating oleogels showed good mechanical stability with a bending radius of ca. 0.18 cm and weight load of 2 kg in the bending (Fig. S6(a)) and compression (Fig. S6(b)) tests, respectively. In addition, a tensile test was conducted to quantitatively estimate the mechanical property of the FAA-incorporating oleogel. In the stress-strain curve of the EPC5.0 gel, a fracture strain of 80 % occurred with the stress of 0.21 MPa (Fig. S6(c)).

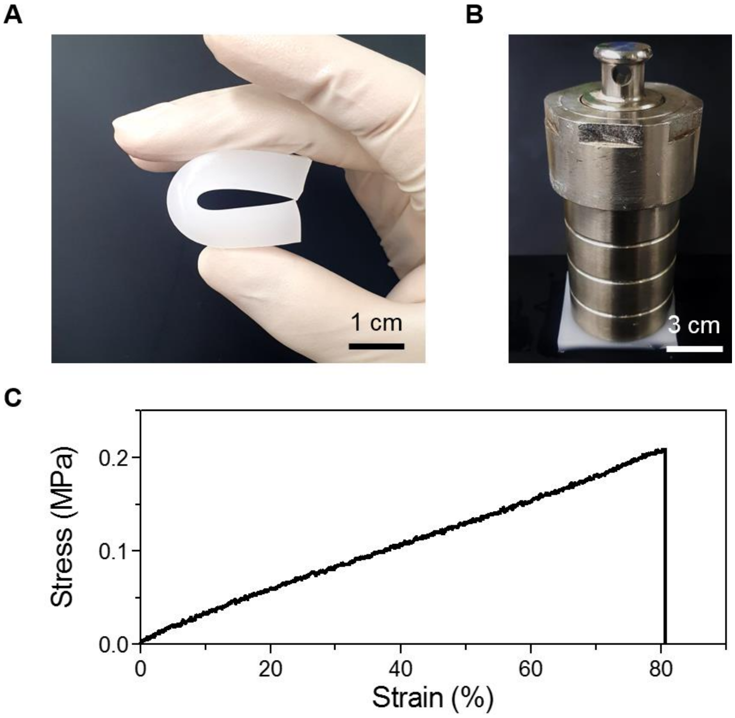

Figure S6. Optical images of (a) bending and (b) compression tests of EPC5.0 gel. (c) Stress-strain curve of the EPC5.0 gel.

S3.6. Molecular content analysis of FAA-incorporating composite surfaces

To analyse the molecular content of FAAs on the FAA-incorporating composite surfaces, EPC5.0 surface was subjected to ToF-SIMS (Time-of-Flight Secondary Ion Mass Spectrometry) analysis. When a focused high-energy ion beam is scanned over a sample surface, mass spectra are obtained by secondary ions emitted from the sample surface. Accordingly, the mass spectra provide spatially-resolved molecular information and a detailed chemical fingerprint of the surface. In the positive ion mode, erucamide atoms were observed at *m/z* 338.34 ($C_{22}H_{44}NO^+$) in the mass spectra (indicated by black arrows in Figs. S7). The characteristic peak of erucamide was detected on EPC5.0 surface, indicating the presence of FAAs on the FAA-incorporating surfaces. The spatial distributions of the erucamide (at *m/z* 338.34) was visualized in the characteristic ion images on the EPC5.0 surface (see Fig. 1d and Fig. 1e in the manuscript).

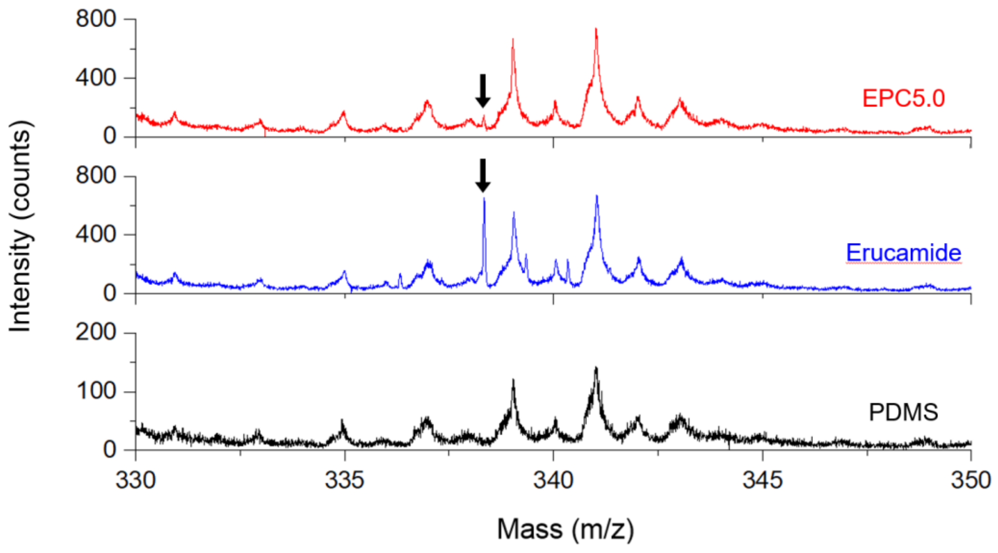

Figure S7. Positive ion Tof-SIMS spectra obtained from PDMS, erucamide, and EPCs with different erucamide content in the mass range from *m/z* = 330 to 350. Characteristic signal from the erucamide is indicated by black arrows (*m/z* 338.34, $C_{22}H_{44}NO^+$).

S3.7. Surface wettability of FAA-incorporating oleogels

The surface wettability of FAA-incorporating oleogels was evaluated by measuring the static contact angle of a water droplet on each surface. The FAA-free PDMS had a water contact angle (CA) of approximately 111°, indicating its hydrophobicity (Fig. S8(a)). EPC and OPC surfaces showed slightly higher and lower CA values, compared with the FAA-free PDMS, respectively. However, both EPCs and OPCs still exhibited hydrophobicity. After hydrophobic silicone oil was impregnated into each surface, the CAs of each oleogel surface were measured after removing an external oil layer on the surface. The overall CAs of the FAA-free (PDMS gel) and FAA-incorporating (EPC gels and OPC gels) oleogels slightly decreased compared to the case of the counterpart solid surfaces (Fig. S8(b)).

In addition, the CA differences among the oleogels with different FAA types (FAA-free, oleamide, and erucamide) indicate the apparent effect of FAAs on the surface chemistry in the lubricant-impregnated gel state, which confirms dual solid (FAA) and liquid (silicone oil) lubricants on the FAA-incorporating oleogel surfaces.

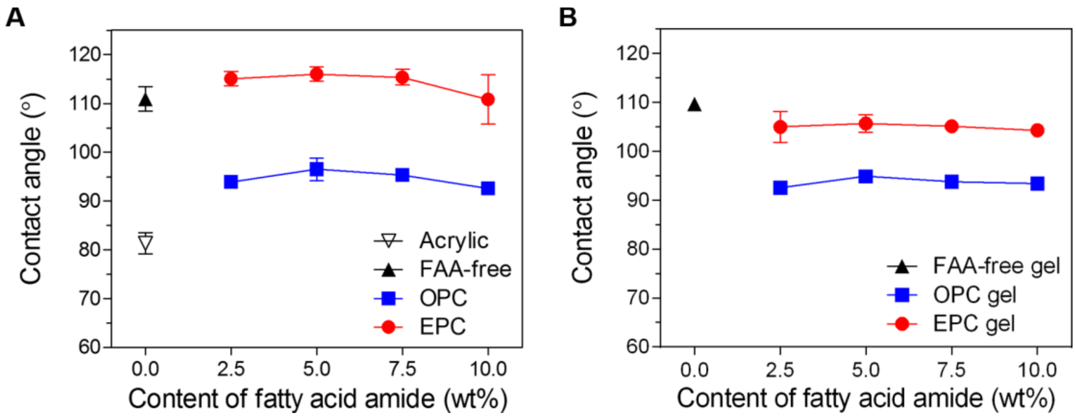

Figure S8. (a) Static water contact angles of a FAA-free PDMS and FAA-incorporating composites (OPCs and EPCs with different FAA content). An acrylic was measured as a control. (b) Static water contact angles of the FAA-free (PDMS gel) and FAA-incorporating (OPC gels and EPC gels with different FAA content) oleogels. 5 μl water droplet was dripped on each surface.

S3.8. Dual penetration of fatty acid amide and oil molecules in PDMS network

In the view that polymeric skeleton dominates mechanical properties of gels, the storage modulus (G′) and loss modulus (G″) values can represent the structural variation of polymer network. Accordingly, the rheological properties of the FAA-free PDMS, FAA-free PDMS gel, EPC5.0, and EPC5.0 gel were measured (Fig. S9). In a wide linear viscoelastic region under oscillatory frequency sweep mode, the G′ values of their solid and gel states were higher than the G″ values, confirming their crosslinked networks.

For the case of the FAA-free polymer network, the PDMS had almost the same storage modulus and a slightly decreased loss modulus upon impregnation of oil into the polymeric network (from dry to gel state). This is attributed to the intrinsically dense polymeric network of the PDMS.

For the case of FAA-incorporating polymer network, the G′ and G″ values of the EPC5.0 were lower than those of FAA-free PDMS due to the presence of erucamide in the polymeric network. Surprisingly, after oil impregnation into the network, the EPC5.0 showed a significant decrease in both G′ and G″ values and an increase in G′/G″ ratio, compared with those variations of the FAA-free PDMS from dry to gel state. The high G′/G″ ratio indicates a more elastic property in the gel state. Accordingly, the polymeric network of the EPC5.0 was more effectively weakened after oil impregnation, compared to the case of the FAA-free PDMS. This higher effect of oil molecules on the structural variation of the EPC polymeric network is related with erucamide chains interpenetrated with the PDMS network, in which the oil distribution becomes more uniform and dense in the crosslinking network. The dense oil distribution in the EPC5.0 gel network was confirmed by its smaller spatial correlation distance between oil molecules and higher oil absorption capacity compared to case of the FAA-free oleogel (see WAXS analysis in Fig. 2f and oil absorption capacity in Fig. 2c of the manuscript).

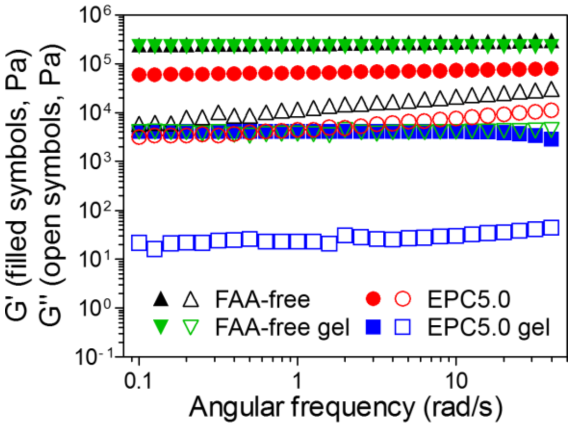

Figure S9. Storage modulus (G′, filled symbols) and loss modulus (G″, open symbols) of the FAA-free (PDMS), EPC5.0, FAA-free oleogel (PDMS gel), and EPC5.0 gel tested in oscillatory frequency sweep mode.

S3.9. Swelling ratio of the wall structure in FAA-incorporating oleogels

To estimate the swelling behavior of wall structure in FAA-incorporating oleogels, the FAA-free oleogel (PDMS gel) was prepared as a control sample. In the view of the excellent solvent compatibility of PDMS, PDMS is amenable to solvent infusion due to its low rotational energy barrier of ca. 3.3 kJ/mol around the $Me_2Si{-}O$ bond (compared with ca. 13.8 kJ/mol around $-CH_2-$ in polyethylene); this low energy barrier allows easy diffusion throughout the polymer matrix.[3] The PDMS become swollen and expanded when immersed in a compatible solvent, such as silicone oil, because polymer chains extend to maximize polymer−solvent interactions.[4] Therefore, the swelling behavior of the wall structure in the FAA-incorporating oleogels could be revealed by the swelling ratio of the PDMS.

For the swelling process of the FAA-free and FAA-incorporating oleogels, silicone oil with a viscosity of 5 cSt was used as a swelling solvent. The EPC5.0 gel film exhibits repeatable shrinking-swelling behaviour with a thickness change between ca. 0.15 mm to ca. 0.26 mm (Fig. S10(a)). The swelling ratio was calculated along the dimension of thickness as follows:

$$\text{Swelling ratio (\%)} = \frac{d_{oil}}{d_{dry}} \times 100$$

where d_{dry} is the initial thickness of a dried state film and d_{oil} is the thickness of an oil-impregnated gel-state film. The film thickness was measured using a profilometer with an accuracy of 0.1 μm. The measured swelling ratios of the FAA-incorporating oleogels (ca. 168 % for the EPC5.0 gel) were higher than that of the FAA-free oleogel (ca. 114% for the PDMS gel) (Figs. S10(b) and 10(c)). Accordingly, these high swelling ratios of the FAA-incorporating oleogels are attributed to the swollen property of the PDMS and additional swollen effect induced by the incorporation of the FAAs.

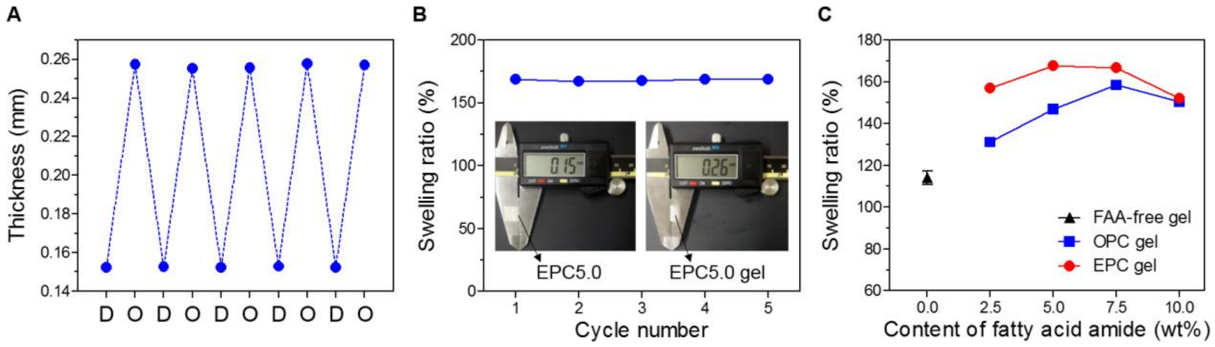

Figure S10. (a) Thickness variations of the EPC5.0 gel in dried (D) and oil-impregnated (O) state. (b) Swelling ratio of the EPC5.0 gel upon the thickness dimension. Inset images show the thickness variation of the film from dry (EPC5.0) to gel (EPC5.0 gel) state. (c) Swelling ratios of FAA-free (PDMS gel) and FAA-incorporating (OPC gels and EPC gels) oleogels upon the thickness dimension.

S3.10. Mechanical property of FAA-incorporating oleogels

The mechanical properties of the FAA-incorporating oleogels were evaluated by tensile stress-strain measurement. To identify effect of the FAAs on mechanical property, the FAA-free (PDMS) and FAA-incorporating composite (OPCs and EPCs) films were evaluated (Figs. S11(a) and S11(b)). The stress at fracture for both OPC and EPC films was ca. 0.3 MPa. The fracture strain of OPCs and EPCs was in the range of ca. 132 % to 201 % and from ca. 133% to 152 %, respectively. The FAA-free PDMS film had a fracture stress of ca. 1.1 MPa and fracture strain of ca. 90 %. Accordingly, the FAA incorporation into the PDMS network resulted in more stretchable polymeric skeleton.

The mechanical properties of FAA-free (PDMS gel) and FAA-incorporating (OPC gels and EPC gels) oleogel films were then evaluated (Figs. S11(c) and S11(d)). The FAA-free oleogel (PDMS gel) film had a fracture stress of ca. 0.45 MPa and fracture strain of ca. 51 %. On the other hand, the stress at fracture for OPC gel and EPC gel films was under ca. 0.16 MPa and under 0.21 MPa, respectively. The fracture strain of FAA-incorporating oleogels was in the range of ca. 57 % to 73 % for OPC gels and from ca. 77 % to 104 %, for EPC gels, resulting in more stretchable gel network than the FAA-free oleogel.

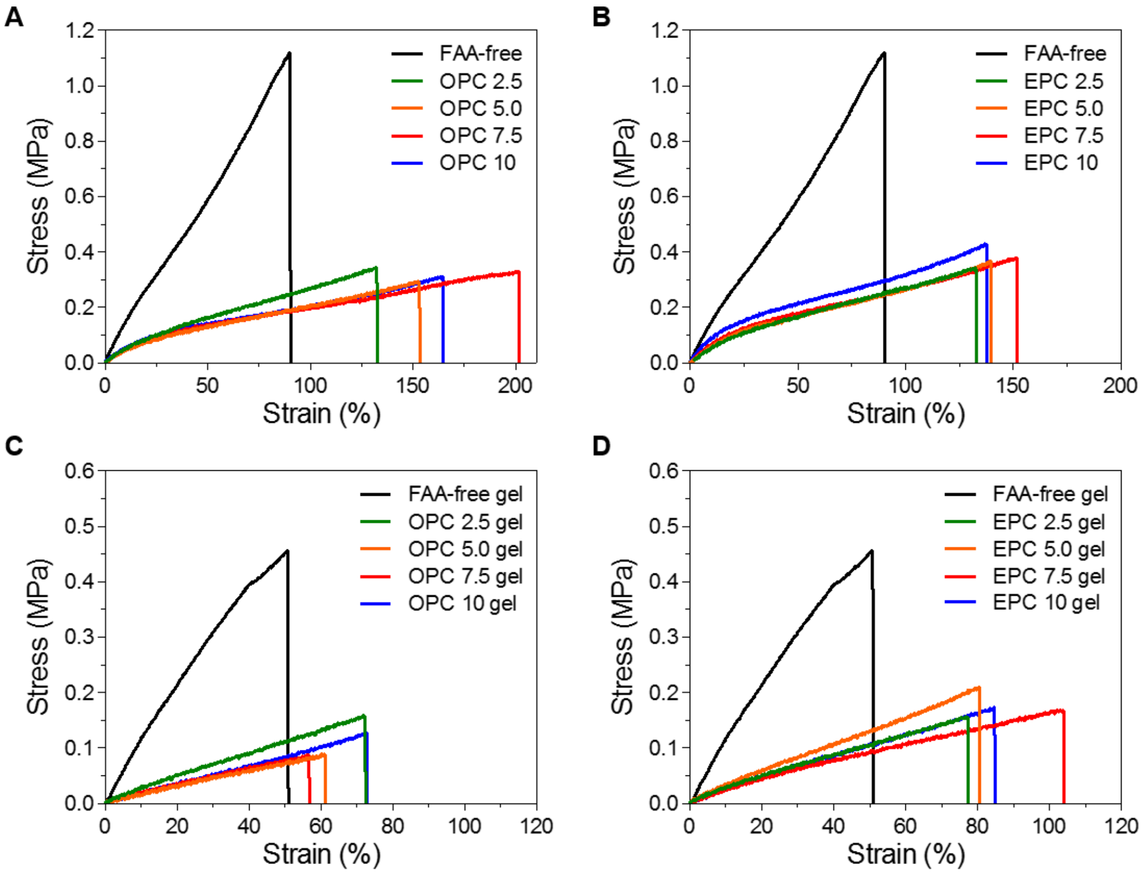

Figure S11. Tensile stress-strain curves of (a) FAA-free (PDMS) and OPCs films with different oleamide content, (b) FAA-free (PDMS) and EPCs films with different erucamide content, (c) FAA-free oleogel (PDMS gel) and OPC gels with different oleamide content, and (d) FAA-free oleogel (PDMS gel) and EPC gels with different erucamide content.

S3.11. Elasticity of FAA-incorporating oleogels

The elastic properties of FAA-incorporating oleogels were evaluated by elastic moduli (Young's moduli) obtained from the tensile stress-strain curves in Fig. S11. The FAA-free PDMS film had an Young's modulus of ca. 1.5 MPa. Both OPC and EPC films had much lower Young's modulus values from ca. 0.4 to 0.8 MPa, compared to the case of FAA-free film (Figs. S12(a) and S12(b)). In addition, the Young's moduli of OPC gels and EPC gels further decreased, which was approximately 3.9~6.9 times smaller than that of a FAA-free oleogel (PDMS gel) (Figs. S12(c) and S12(d)). Accordingly, the FAA-incorporating oleogels exhibits more elastic properties than the FAA-free oleogel.

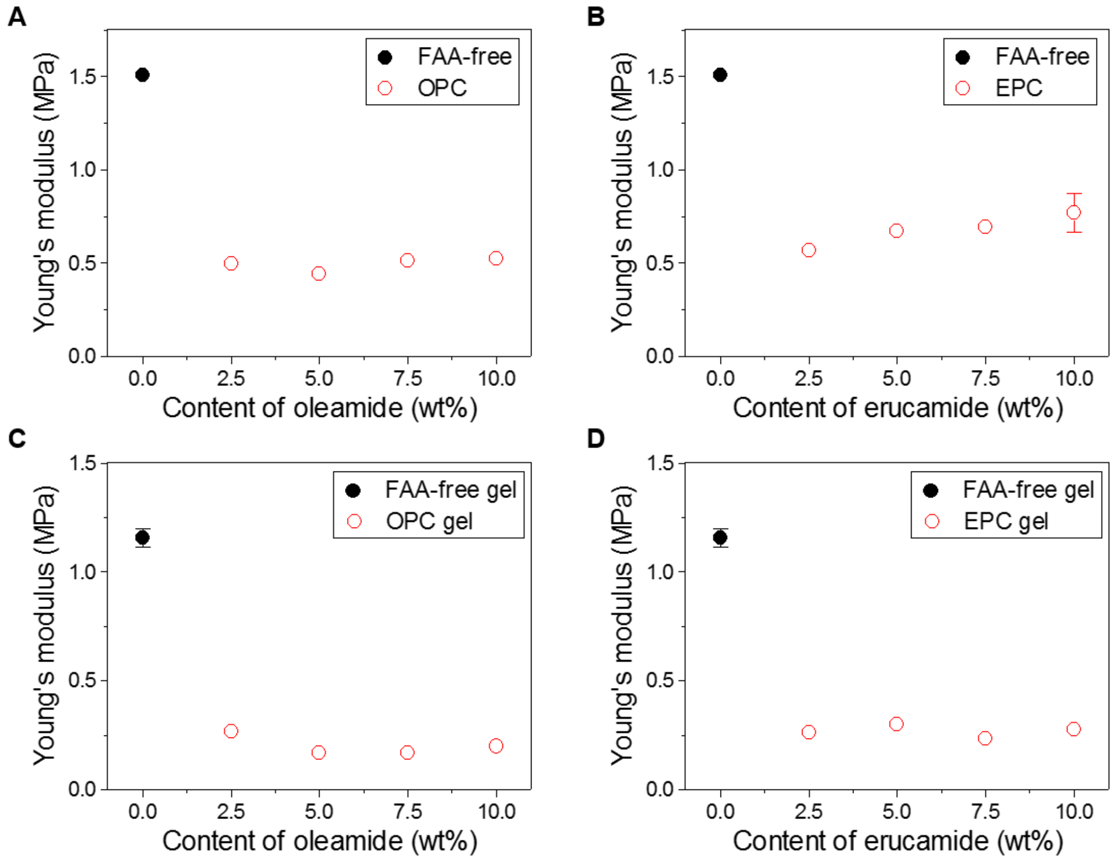

Figure S12. (a, b) Young's moduli of the FAA-free PDMS and FAA-incorporating composites (OPCs and EPCs). (c, d) Young's moduli of the FAA-free (PDMS gel) and FAA-incorporating (OPC gels and EPC gels) oleogels.

S3.12. Water repellence of FAA-incorporating oleogels

To estimate the water repellence of FAA-incorporating oleogels, dynamic contact angles (CA) and sliding angles of a water droplet on the tilted FAA-incorporating oleogels were measured. When a water droplet slides on each surface, advancing CA and receding CA of the sliding water droplet were measured. Contact angle hysteresis (CAH) is the difference between the measured advancing and receding CAs of a moving water droplet, which is qualitatively related with droplet mobility against resistance.[9] Sliding angle is the tilted angle of a surface required for moving a water droplet on it. Low values of CAH and sliding angle indicate a liquid-repellent surface with little pinning of droplets.[9]

To confirm the lubrication effect of FAAs as slip agents, CAH and sliding angle were measured for FAA-free and FAA-incorporating surfaces. The CAH values of the OPCs and EPCs were approximately 1.9~2.4 times lower than that of the FAA-free PDMS (Figs. S13(a) and S13(b)). The low CAH values of the OPCs and EPCs resulted in low sliding angle of a water droplet (Fig. S14(a)). Accordingly, the low CAH and sliding angle of OPCs and EPCs demonstrated the lubricant property of FAAs.

In addition, the FAA-free and FAA-incorporating oleogels showed lower CAH and sliding angle than the counterpart FAA-free and FAA-incorporating solid surfaces (Figs. S13(c), S13(d), and S14(b)). In particular, the FAA-incorporating oleogels (OPC gels and EPC gels) exhibited lower CAH and sliding angles than the FAA-free oleogel (PDMS gel), indicating the high water repellence of FAA-incorporating oleogels. Accordingly, the water repellence of the FAA-incorporating oleogels was amplified by integrating FAAs and oil molecules in the hybrid configuration.

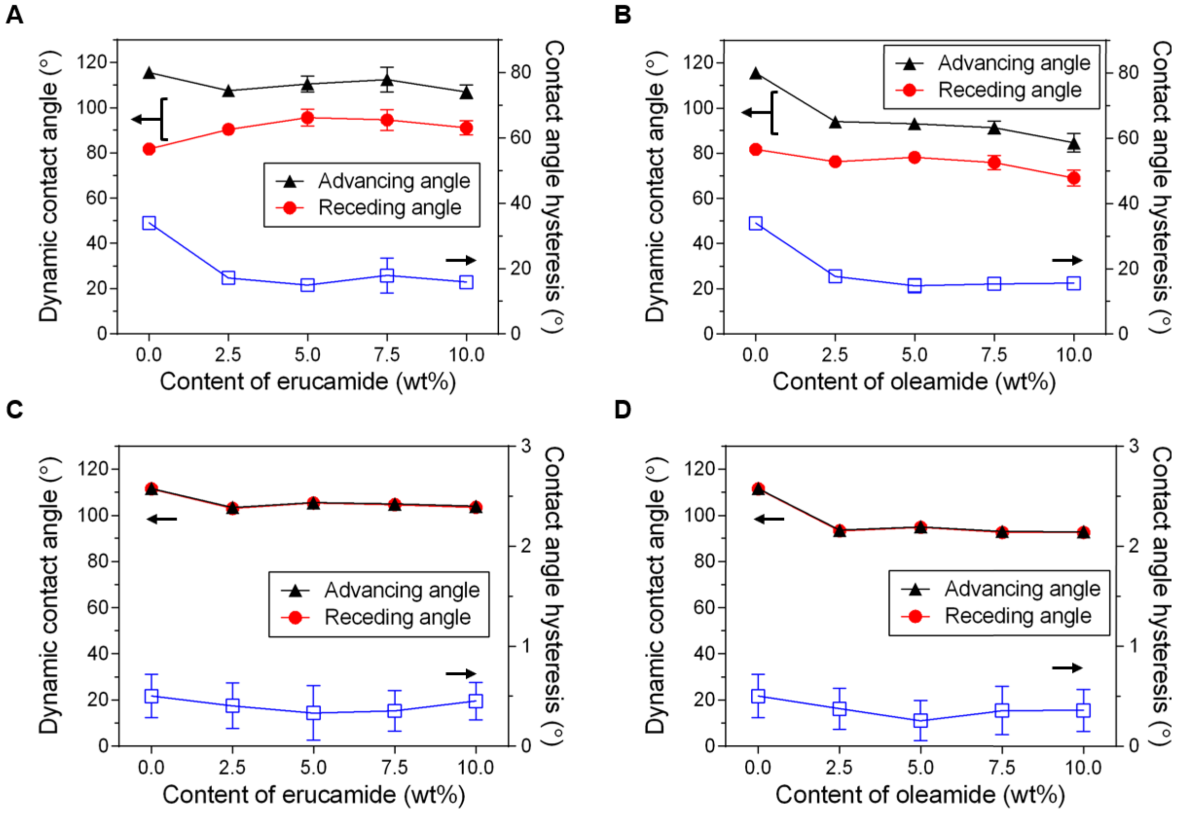

Figure S13. Dynamic water contact angle (advancing/receding angles, filled symbols, left y-axis) and contact angle hysteresis (blue open symbols, right y-axis) of (a) EPCs, (b) OPCs, (c) EPC gels, and (d) OPC gels. FAA-free PDMS and FAA-free PDMS gel were measured as controls. 10 μl water droplets were dripped on each surface.

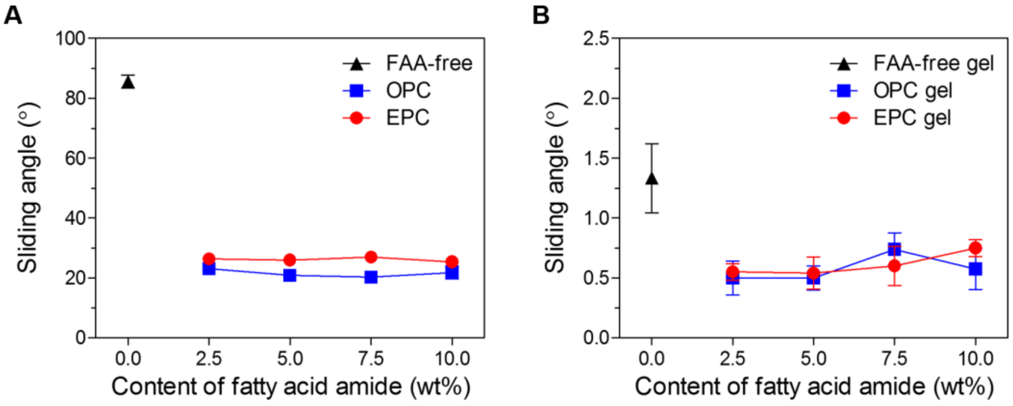

Figure S14. Water sliding angle of (a) FAA-free PDMS and FAA-incorporating composites (OPCs and EPCs), and (b) FAA-free (PDMS gel) and FAA-incorporating (OPC gels and EPC gels) oleogels. 10 μl water droplets were dripped on each surface.

S3.13. Solubility of FAA for silicone oil

The solubility of FAA for silicone oil was evaluated by mixing 3g of oleamide powder with 10ml of 5 cst silicone oil (Fig. S15(a)). The mixed oleamide powder with silicone oil was stored at room temperature (Fig. S15(a-i)) and in a 60°C oven (Fig. S15(a-ii)) for 1 day. Even after 1 day storage in a 60°C oven, the oleamide powder was barely dissolved in silicone oil, indicating extremely low solubility of FAA in silicone oil.

The solubility of FAA in silicone oil was further investigated by impregnating a bare PDMS with the silicone oil containing oleamide powder. After 1-day storage in Fig. S6(a-ii), the silicone oil mixed with oleamide was impregnated into the bare PDMS. The surface characteristics of the PDMS impregnated with the mixed silicone oil was evaluated by measuring its water contact angle (Fig. S15(b)) and sliding angle (Fig. S15(c)). The PDMS impregnated with the mixed silicone oil (red-dotted line in Fig. S15(b, c) showed similar water contact angle and sliding angle with those of the FAA-free PDMS oleogel (black triangular symbol in Fig. S15(b, c)). This result indicates that the solubility level of FAA in silicone oil does not affect the surface characteristics and slippery properties of the gel. In other words, the low solubility of FAA in silicone oil implies that the enhanced surface properties of the FAA-incorporating oleogel were not induced by a result of dissolution of FAAs in silicone oil of the gel system. In addition, the low solubility of FAA in silicone oil reduces the leaching risk of FAA to the surrounding bulk water even though oil loss occurs under shear flow.

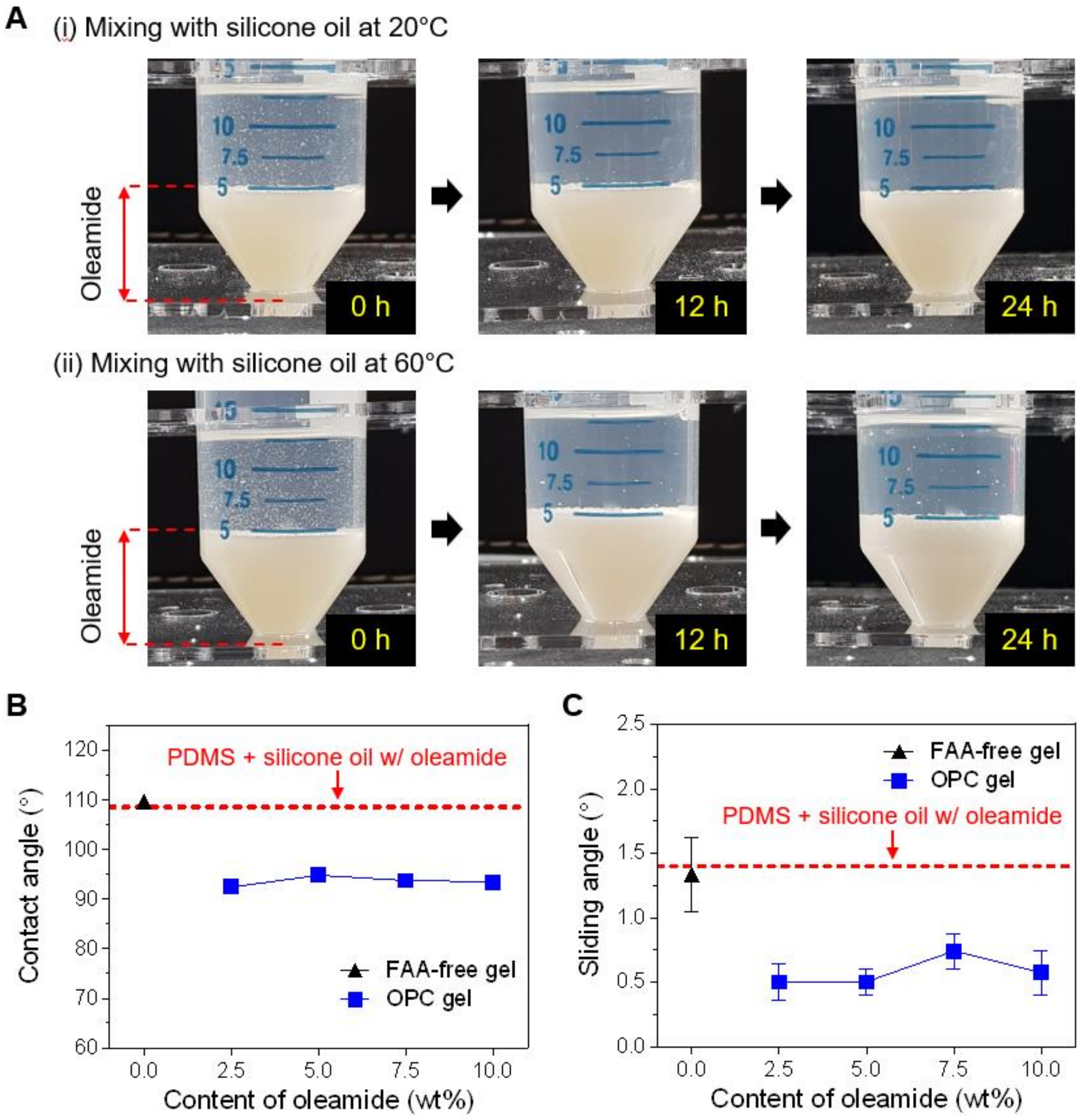

Figure S15. (a) Solubility of oleamide in silicone oil (5 cst). The oleamide was mixed with silicone oil (i) at room temperature and (ii) in a 60°C oven for 1 day. After 1 day storage at 60°C oven, the mixed silicone oil in Fig. S6 (a-ii) was used for the organogelation of a bare PDMS film. The resulting PDMS impregnated with silicone oil containing oleamide was tested for measuring water contact angle and sliding angle on the surface. (b) Static water contact angle of the PDMS impregnated with silicone oil containing oleamide (indicated by red dotted line). 5 µl water droplet was dripped on the surface. (c) Sliding angle of the PDMS impregnated with silicone oil containing oleamide (indicated by red dotted line). 10 µl water droplet was dripped on the surface."

S3.14. Slip flow over FAA-incorporating oleogel surfaces

The highly slippery feature of FAA-incorporating oleogels enables slip phenomena on its surface. To prove the slip phenomena on the oleogel surface, the velocity field over the oleogel surface was visualized. The oleogel surface was placed on the bottom of a channel (10mm wide and 10mm high) and tracing particles were seeded in the working fluid flowing at a Reynolds number of 50 in the channel (Fig. S16(a)). At a fully-developed region, velocity profiles of the flow in the channel center and near-wall regions were measured by using particle image velocimetry (PIV) and particle tracking velocimetry (PTV) techniques, respectively. The measured streamwise velocity (u) was normalized by the time-averaged maximum velocity (u_{max}). The normalized streamwise velocity profile according to the normalized height of the channel is shown in Fig. S16(b). The velocity profile shows that no slip velocity occurs on a bare flat surface at y/H=1 with the satisfaction of the no-slip boundary condition. However, in the normalized streamwise velocity profile, the finite velocity at y/H=0 (gel/water interface) confirms the presence of slip length at the gel surface. The manified velocity profile in the vicinity of the oleogel surface (y/H=0) is illustrated in the inset of Fig. S16(b). This result supports the slip flow on the surface of FAA-incorporating oleogels.

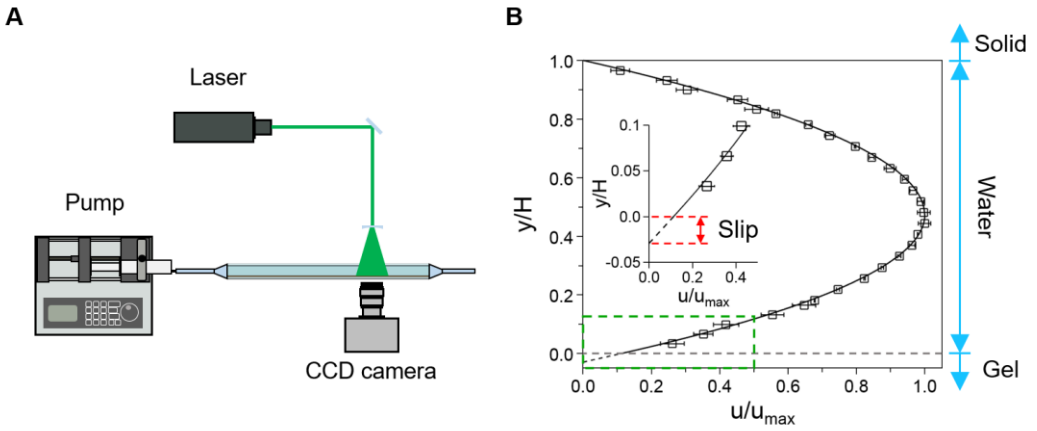

Figure S16. (a) Experimental setup for the visualization of slip flow over FAA-incorporating oleogels. (b) Normalized streamwise velocity (u/u_{max}) profile of the flow over OPC5.0 gel. Inset graph is the magnified velocity profile for a green-dotted box, showing the slip length marked below the surface (y/H=0). H: channel depth, u_{max}: maximum streamwise velocity.

S3.15. Liquid repellence of FAA-incorporating oleogels

FAA-incorporating oleogels exhibited highly slippery properties against water. Water is a low viscous fluid that has a low resistance to shear forces and is easy to move molecules. In addition, the FAA-incorporating oleogels were repellent to highly viscous liquid such as honey, non-Newtonian liquids such as ketchup and honey, and milk (Fig. S17). Accordingly, the FAA-incorporating oleogels exhibited high liquid repellence.

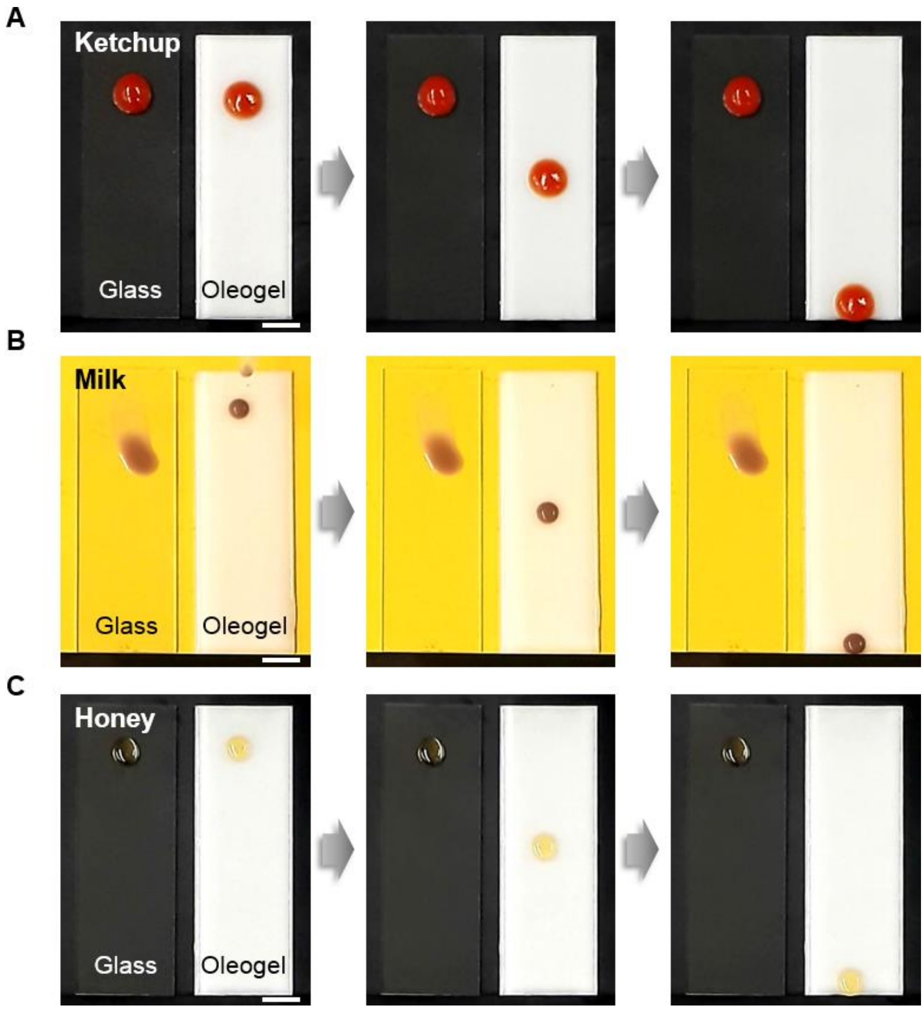

Figure S17. Sliding motion of (a) ketchup, (b) milk, and (c) honey droplets on a tilted bare glass and EPC5.0 gel surface. The tilted angle was approximately 5°. The white-coloured scale bars represent 1 cm.

S3.16. Experimental setup of a high-speed water cavitation tunnel test

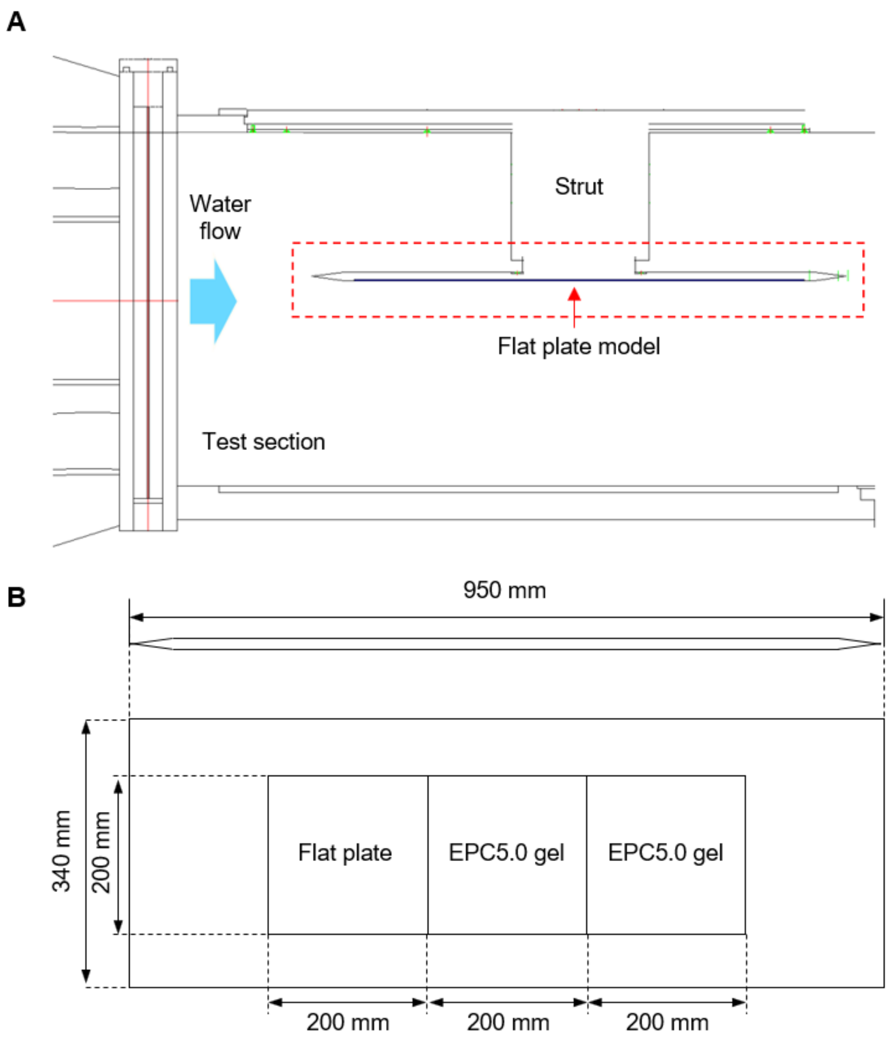

Figure S18. Experimental setup used for shear-stability test in a cavitation tunnel. (a) A flat plate model with a leading edge was installed in the cavitation tunnel. (b) Two EPC5.0 gel-coated surfaces (20 cm width × 20 cm length) were mounted on the flat plate model (red dotted box in Fig. S14(a)).

S3.17. Anti-bacteria property of FAA-incorporating oleogels

To evaluate the antifouling and antimicrobial activity of FAA-incorporating oleogels, Gram-negative *E. coli* (*Escherichia coli*) bacteria were cultured on various oleogels and control surfaces (Fig. S19(a)). After 24 h incubation, *E. coli* cultured on EPC surfaces (Fig. S19(a-ii)) showed decreased adhesion compared to that grown on a FAA-free surface (PDMS, in Fig. S19(a-i)). In addition, OPC surfaces showed decreased adhesion as well (Fig. S19(a-iii)). Those low adhesion of *E. Coli* on the EPC and OPC surfaces demonstrates the intrinsic antifouling nature of FAAs (oleamide and erucamide). The anti-biofouling properties of FAAs were quantitatively confirmed through the coverage area of the attached bacteria (Fig. S19(b)). The EPCs and OPCs with higher FAA content exhibited higher anti-microbial activity against *E. coli*. *E. coli* cultured on the solid surfaces (PDMS, OPCs, and EPCs) were alive and dead in similar proportions, which is indicated by the emitted green (live) and red (dead) fluorescence.

When the solid surfaces were organogelated through the impregnation of silicone oil, the bacterial adhesion on the oleogels was significantly reduced compared to the case of the counterpart solid surfaces (Fig. S19(a-iv, v, and vi)). It is notable that *E. coli* on a FAA-free oleogel (PDMS gel) were all alive, as evidenced by the green fluorescence emitted (Fig. S19(a-iv)). This indicates that oleogels suppress the bacterial attachment with its antifouling capability, rather than killing the bacteria. The impregnated silicone oil in the oleogels is non-fluorinated hydrophobic lubricant with non-toxicity, while most reported slippery liquid-infused porous surfaces (SLIPS) used fluorinated lubricants such as DuPont krytox oils and 3M fluorinert FC-70 whose perfluoroalkyl building blocks can induce toxicity issue and ecological impacts[22, 23].

In particular, the FAA-incorporating oleogels (EPC gels and OPC gels) exhibited higher antifouling effects than the FAA-free oleogel. Accordingly, the anti-biofouling performance

could be amplified by integrating FAAs and oil molecules in the hybrid configuration. In particular, all the FAA-incorporating oleogels with different FAA content exhibited zero-attachment of bacteria on their surfaces, which demonstrates their superior antibiofilm formation capability.

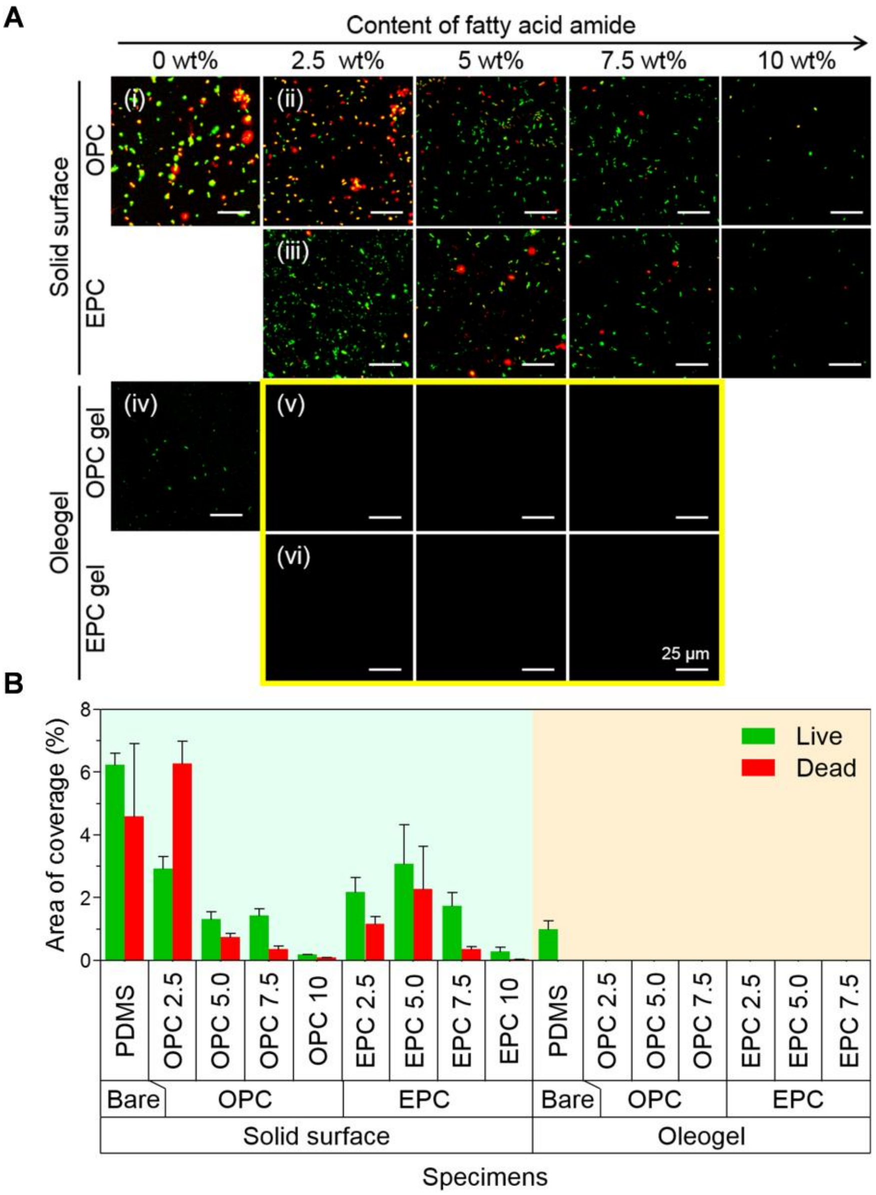

Figure S19. (a) Confocal microscopy images of *E. coli* cultured on (i) FAA-free (PDMS), (ii) OPCs, (iii) EPCs, (iv) FAA-free oleogel (PDMS gel), (v) OPC gels, and (vi) EPC gels for 24 h. The bacteria were stained with a fluorescent labelling agent (live/dead bacterial viability

kit). Live cells emit green light and dead cells emit red light. The scale bar represents 25 μm. (b) Quantification of coverage area of the live (green) and dead (red) cells cultured on the samples.

S3.18. Anti-marine bacteria property of FAA-incorporating oleogels

To assess the anti-biofouling activity of FAA-incorporating oleogels for practical marine application, marine bacteria *Maribacter dokdonensis* (*M. dokdonensis*) are cultured on various gels and control surfaces. After 24 h incubation, the coverage areas of *M. dokdonensis* cultured on EPCs (Fig. 20-ii) and OPCs (Fig. 20-iii) were about 1.4~5.6 times smaller than that on a bare PDMS surface (Fig. 20-i)). After organogelation, the bacterial adhesion on oleogels (Fig. 20-iv, v, and vi) is significantly reduced compared to the counterpart solid surfaces. Compared to the coverage area (0.5~2.5%) of M. dokdonensis on FAA-free oleogel, the FAA-incorporating oleogels with FAA content over 5wt% exhibit zero-attachment of bacteria on their surfaces.

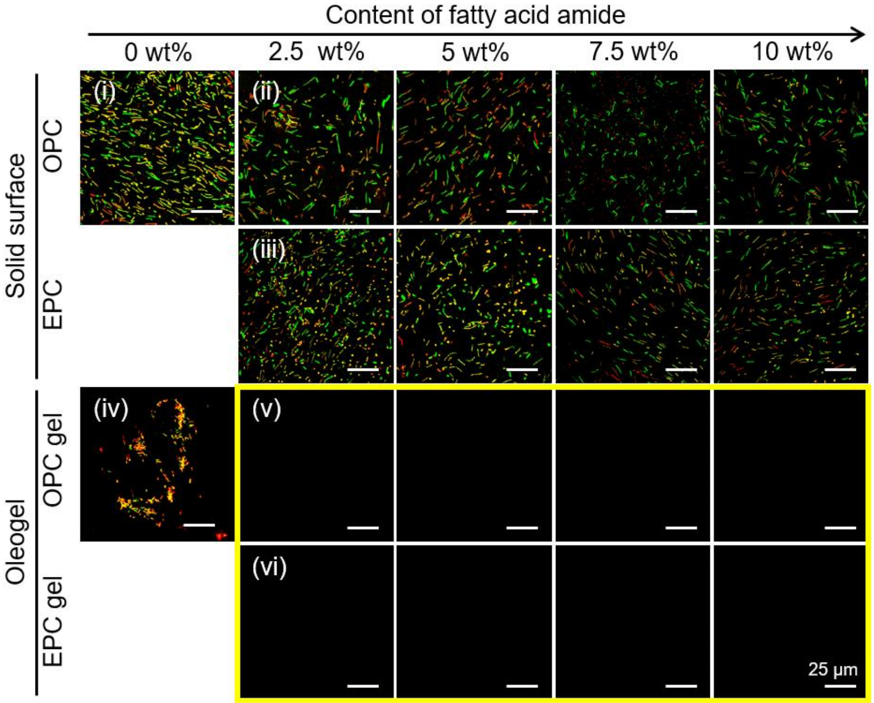

Figure 20. Confocal microscopy images of *Maribacter dokdonensis* (*M. dokdonensis*) cultured on the (i) FAA-free (PDMS), (ii) OPCs, (iii) EPCs, (iv) FAA-free oleogel (PDMS gel), (v) OPC gels, and (vi) EPC gels with different FAA content for 24 h. The marine bacteria were stained with a fluorescent labeling agent (live/dead bacterial viability kit). Live cells show green color and dead cells show red color. The scale bar represents 25 μm.

S3.19. Anti-brown algae bioassay experiment

Haploid gametophyte brown algae (*Cladosiphon sp*) were cultured on various oleogels and control surfaces to assess the anti-biofouling property of FAA-incorporating oleogels (Fig. S21(a)). After 24 h incubation, brown algae cultured on both EPC and OPC surfaces were sharply less than those grown on a FAA-free surface (PDMS). These low adhesion of brown algae on the EPCs and OPCs demonstrates the intrinsic antifouling nature of FAAs. The anti-biofouling properties of FAAs were quantitatively confirmed through the coverage area of the attached algae (Fig. S21(b)). The EPCs and OPCs with higher FAA content exhibited higher anti-biofouling property against brown algae.

After organogelation, the algae adhesion on the FAA-free and FAA-incorporating oleogels was slightly reduced compared to the case of the counterpart FAA-free and FAA-incorporating solid surfaces (Fig. S21(a)). In particular, the reduction rates of brown algae upon oil impregnation from dry to gel state (= the ratio of the biofilm area on solid surfaces to the biofilm area on gel surfaces) were relatively small, compared to the case of *E. coli* and maribacter (*M. dokdonensis*), indicating less biofouling effect of oil molecules for the brown algae. The coverage area of brown algae on the FAA-free oleogel (PDMS gel) was approximately 33.0 %, which shows the limited anti-algal activity of previous oleogels (rely on liquid lubricant). Notably, the coverage area (ca. 33.0 %) of brown algae on the FAA-free oleogel was much higher than those (ca. 0.9~2.0 %) on the FAA-incorporating solid composites (OPCs and EPCs), which demonstrates that the brown algae activity is majorly suppressed by FAAs rather than oil molecules.

The FAA-incorporating oleogels (EPC gels and OPC gels) exhibited much higher antifouling effects than the FAA-free oleogel. The coverage area of brown algae on the EPC5.0 gel was only ca. 1.2 %. Accordingly, the antifouling capability for brown algae was mainly affected by the intrinsic biofouling property of FAAs. These results demonstrate that

the dual solid (FAAs) and liquid (oil) lubricants-based gel system is required for excellent anti-algae property, compared to the conventional liquid lubricant-based gel system.

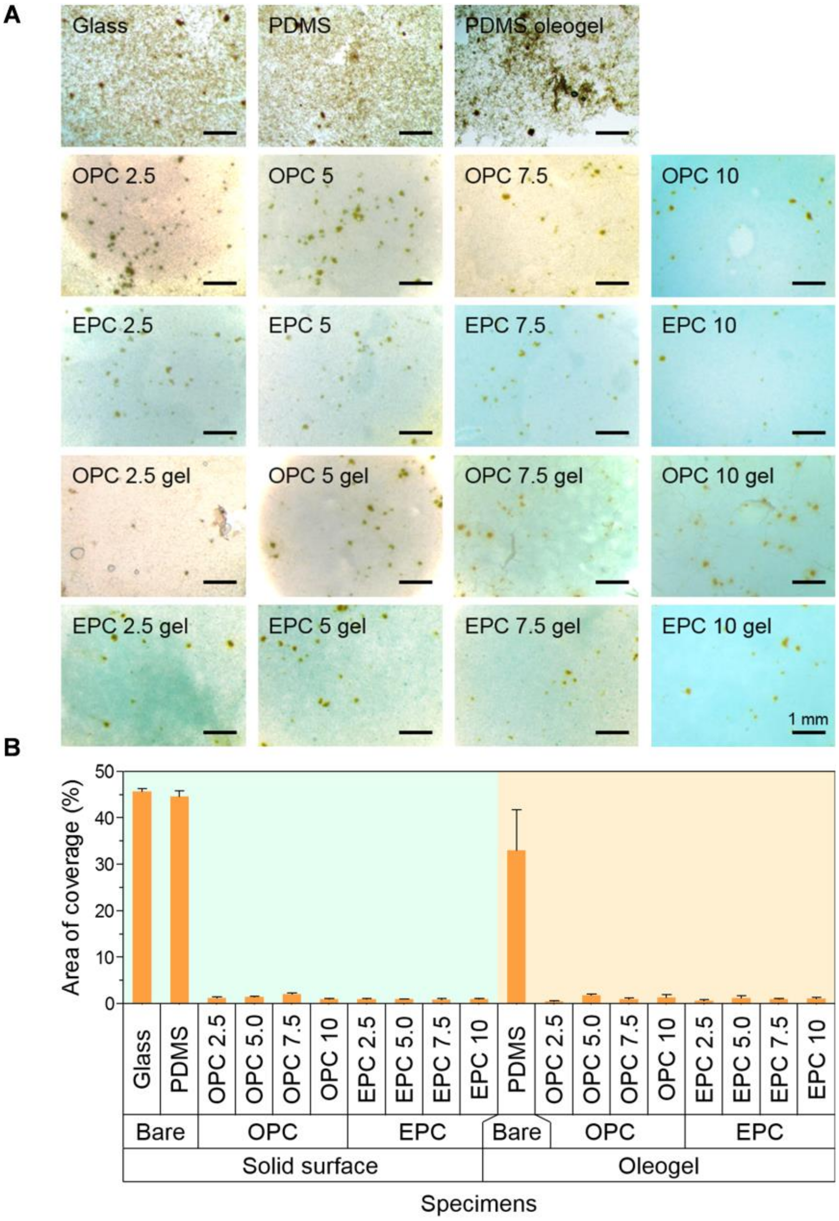

Figure S21. (a) Microscopic images of brown algae (*Cladosiphon sp*) cultured on a glass, FAA-free PDMS, OPCs, EPCs, FAA-free oleogel (PDMS gel), OPC gels, and EPC gels for 24 h. The scale bar represents 1 mm. (b) Quantification of coverage area of brown algae cultured on the test samples.

S3.20. Optimum surface energy of FAA-incorporating oleogels for anti-bioadhesion

The surface energy of FAA-incorporating oleogels was estimated from measured static contact angles (CA) (Supplementary Fig. S8) and water surface tension as follows:

$$\mathbf{cos\theta = -1 + 2\sqrt{\frac{\gamma_s}{\gamma_l}}[1 - \beta(\gamma_L - \gamma_S)^2]}$$

where θ denotes the water contact angle, γ_s is the surface energy of a substrate, γ_l is the surface energy of water (72.8 mJ/m^2), β is a constant value of 1.057 × 10^{-4} m^2/mJ.[10] Based on the above equation, a substrate with a high CA has low surface energy. The low surface energy can be related to low adhesion for marine biofoulants.[11] The FAA-free (PDMS) and FAA-incorporating composites (EPCs and OPCs) had low surface energy due to their hydrophobicity, compared to case of a hydrophilic acrylic surface (Fig. S22(a)).

After organogelation, the surface energy range of the FAA-incorporating oleogels (EPC gels and OPC gels) was from ca. 19.7 to 27.3 mJ/m^2 (Fig. S22(b)). The FAA-free oleogel (PDMS gel) had surface energy of ca. 17.5 mJ/m^2. According to the Baier curve (bioadhesion curve versus surface free energy in the range of 10 to 70 mJ/m^2), minimum bioadhesion did not occur at the lowest surface energy, but occurred in the optimum energy range (from ca. 20 to 30 mJ/m^2).[12] The surface energy values of the EPC gels and OPC gels belong to the optimum surface energy range for the minimum bioattachment. Accordingly, in terms of surface energy, the FAA-incorporating oleogels are more suitable for anti-biofouling than the FAA-free oleogel.

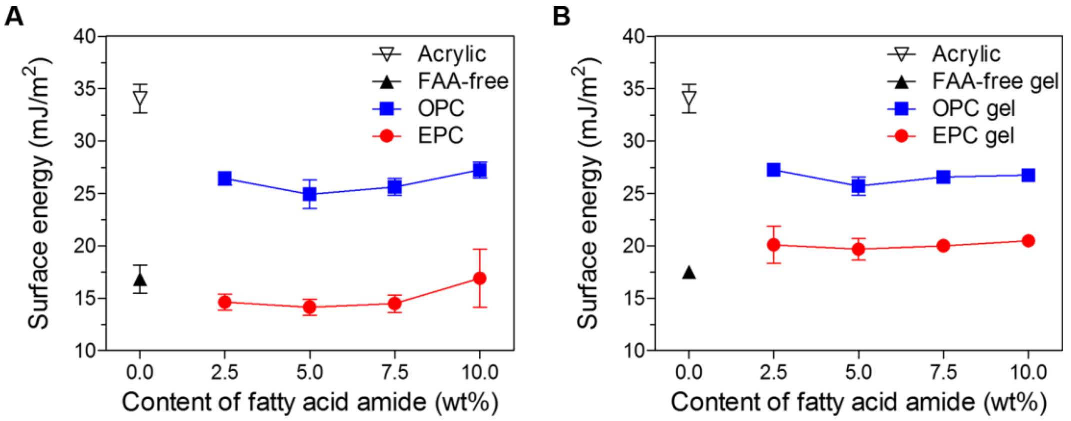

Figure S22. Surface energy of (a) FAA-free (PDMS) and FAA-incorporating (EPCs and OPCs) composites and (b) FAA-free (PDMS gel) and FAA-incorporating (EPC gels and OPC gels) oleogels. An acrylic was estimated as a control.

S3.21. Low pull-off force of FAA-incorporating oleogels

The cooperation effects of low elastic modulus and low surface energy of FAA-incorporating oleogels on anti-biofouling performance can be evaluated by Griffth's theory of rupture as follows:

$$F = \sqrt{\frac{2E\gamma}{A\pi}}$$

where F is the stress at fracture, A is the flaw length, E is the Young's modulus, and γ is the surface energy density.[13] From the above equation, $F\sqrt{A\pi}$ indicates a pull-off force required to separate foulants from the surface. The pre-measured and calculated surface energy (Supplementary Fig. S22) and Young's modulus (Supplementary Fig. S12) of each oleogel sample were used for the calculation of each pull-off force.

The FAA-incorporating composites (OPCs and EPCS) had lower pull-off forces than FAA-free surface (PDMS) (Fig. S23(a)). The pull-off force of EPC5.0 was approximately 1.6 and 107 times lower than those of the FAA-free PDMS and acrylic surface, respectively. After organogelation, the pull-off force of EPC gels and OPC gels further decreased (Fig. S23(b)). The pull-off force of EPC5.0 gel was approximately 1.8 and 136 times lower than those of the FAA-free oleogel (PDMS gel) and acrylic surface, respectively. These low pull-off forces of FAA-incorporating oleogels help to mitigate the adhesion of foulants and prevent biofouling. In particular, those effect of low pull-off force on anti-biofouling could be maximized in marine environment with high turbulence intensity, where turbulent flow give rise to a variety of fluid/solid interactions.

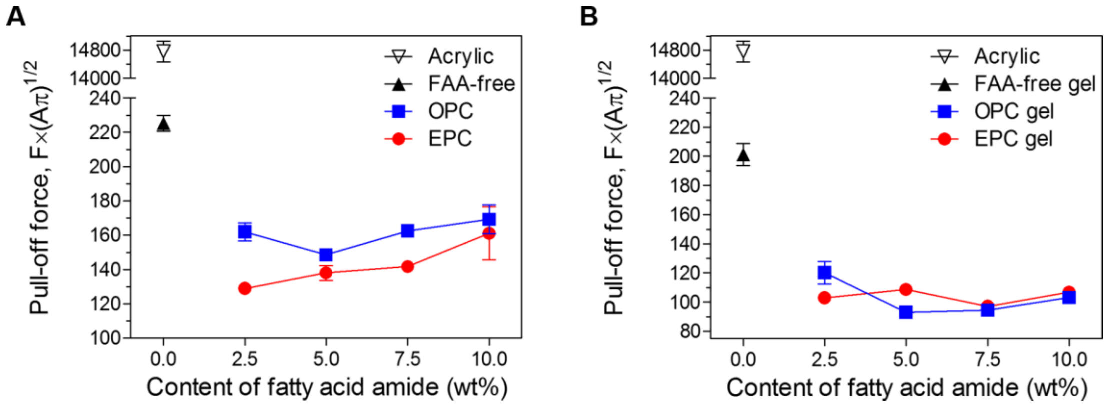

Figure S23. Pull-off forces of (a) FAA-free (PDMS) and FAA-incorporating (EPCs and OPCs) composites and (b) FAA-free (PDMS gel) and FAA-incorporating (EPC gels and OPC gels) oleogels. An acrylic was estimated as a control.

S3.22. Long-term marine filed test near sea farm

The long-term marine field test was conducted in the Yellow Sea at the latitude of 36°08'12.5"N and longitude of 126°32'27.4"E near the Korean city of Seocheon (detailed experimental conditions in Supplementary Methods). The field test place was located next to a seaweed farm (kelp farm), where marine organisms and seaweeds are easily attached to the surfaces. Test samples were mounted in a frame and immersed in seawater at a depth of ca. 1.5 m from sea level (Fig. S24(a)). In other words, a hydrostatic pressure of ca. 1.160 bar was applied to the test samples during the long-term field test.

The long-term field test was conducted for 11 weeks (from 25 March 2020 to 8 June 2020). The acrylic, FAA-free PDMS, OPCs, EPCs with different FAA content were installed in 'A' region (Fig. S24(a, b)). The FAA-free (PDMS gel) and FAA-incorporating (OPC gels and EPC gels with different FAA content) oleogels were installed in 'B' region (Fig. S24(a, c)).

After 11 weeks, marine organisms and seaweed were heavily deposited on the solid surfaces (Fig. S15(a)). To identify biofouling environmental conditions in the field test, the marine biofouling organisms attached on the solid surfaces were analysed. The various biofilm composites were observed including marine bacteria, diatoms, and filamentous red algae (Figs. S26(a) and (b)). Under this extreme biofouling condition, EPC and OPC surfaces exhibited relatively higher biofouling performance than the acrylic and FAA-free PDMS surfaces (see Figs. S25(b, c)). All the EPCs (Fig. S25(b)) and OPC2.5 (Fig. S25(c)) coated on acrylic plates were detached from the substrate due to the adhesive problem. However, the undetached OPC surfaces (5.0, 7.5, 10 wt%) showed relatively clean compared to the acrylic and FAA-free PDMS surfaces, confirming the long-term anti-biofouling property of FAAs (Fig. S25(c)).

Surprisingly, the FAA-incorporating oleogels showed high anti-biofouling properties with preventing the attachment of marine organisms ('B' region in Fig. S19(a), and Fig. S25(d)).

The overall FAA-incorporating oleogels (EPC gels and OPC gels) remained their clean surfaces compared to the FAA-free oleogel (PDMS gel), which indicates the durable lubrication property of the FAA-incorporating oleogels in the marine environment. For the case of OPC10 gel, the OPC10 gel film was detached from the aluminium substrate, which results in foulant attachment on the substrate. In particular, the EPC5.0 gel exhibited the highest anti-biofouling performance with little biofilm attachment, demonstrating its outperforming sustainability of the anti-biofouling property.

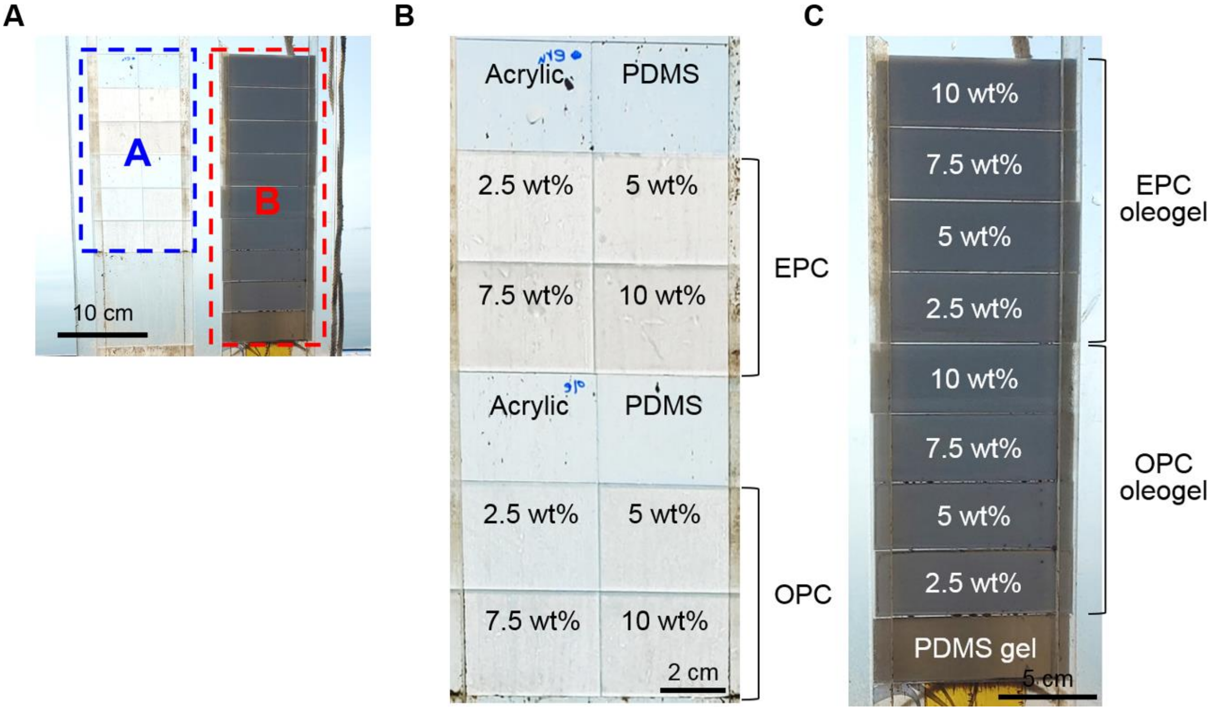

Figure S24. (a) Optical image of a frame in which test samples were mounted for marine field test on 25 March 2020. 'A' region (blue dotted box): solid samples (acrylic, FAA-free PDMS, OPCs, and EPCs), 'B' region (red dotted box): oleogel samples (FAA-free PDMS gel, OPC gels, and EPC gels). (b) Magnified image of 'A' region, showing acrylic, FAA-free PDMS, OPCs, and EPCs. (c) Magnified image of 'B' region, showing FAA-free (PDMS gel) and FAA-incorporating (OPC gels and EPC gels) oleogel-coated aluminium plates.

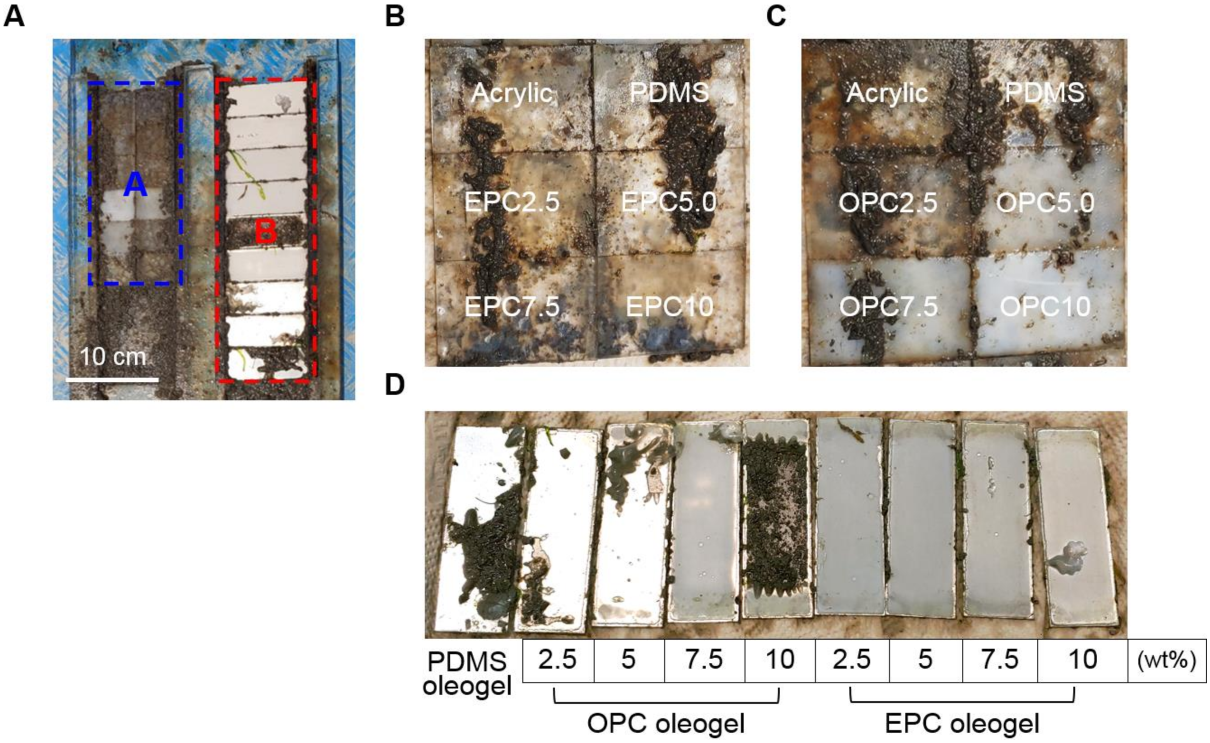

Figure S25. (a) Optical image of the frame after 11 weeks field test on 8 June 2020. (b, c) Optical image of solid samples in the 'A' region. The solid samples (all the EPCs and OPC2.5) coated on the acrylic were detached from the substrate, resulting in marine biofouling on the substrate. (d) Optical image of oleogel samples in the 'B' region. The OPC10 gel coated on the aluminium plate was detached from the substrate, resulting in marine biofouling on the substrate.

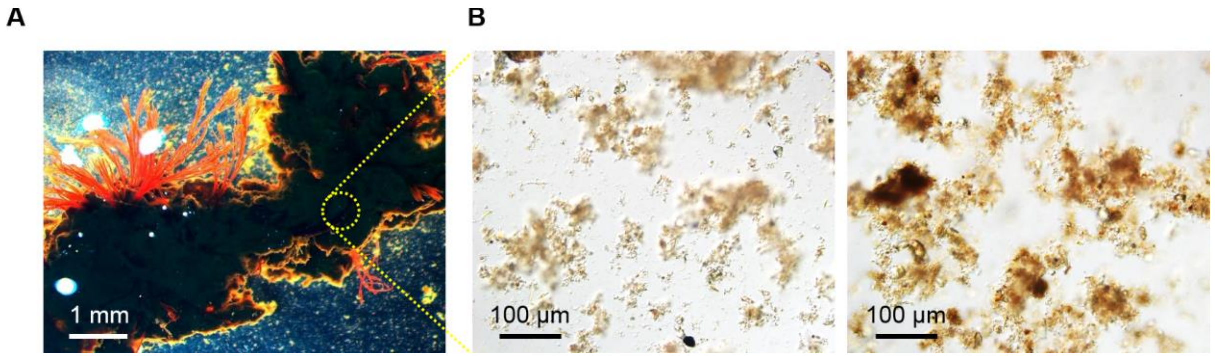

Figure S26. (a) Stereomicroscope image of marine biofouling organisms (biofilm with filamentous red algae) attached to the surfaces. (b) Magnified microscopic images of the yellow-dotted circle region in Fig. S20(a), showing biofilm composites with marine bacteria and diatoms.

S3.23. Long-term marine field test attached to an operating ship

The long-term marine field test using a ship was conducted at west sea located at the latitude of 36°08'12.5"N and longitude of 126°32'27.4"E near the Seocheon town in Korea. The samples were attached to the bottom of a commercial FRP ship (fiber reinforced plastic ship, 8.1 m in length, 3.63 m in width, 0.88 m in height, 3.56 ton in weight). The FAA-free PDMS, OPCs, and EPCs with different FAA content were prepared with the size of 20 × 20 cm^2 as control groups. When the ship was floated on the sea, the underwater distance between the attached samples and sea level was approximately 0.3 m. In other words, a hydrostatic pressure of 1.0427 bar was applied to the samples during a long-term field test. The salinity and temperature of seawater were ranged from 31.5 to 33.0 ppt and from 6.8 to 24.3 °C, respectively. The pH of seawater was 8.1±0.1.

The ship operated for an average of 6 h daily. During the operation time, the minimum and maximum velocity of the ship was 18.5 and 55.6 km/h, respectively. The average velocity was 27.8 km/h. After the operation, the ship was floated on the shore. For the control samples (FAA-free PDMS, EPCs, OPCs), the field test was conducted for approximately 1 month (from 1 May 2020 to 8 June 2020) (Figs. S27(a-d)). For the EPC5.0 gel-coated surfaces, the field test was conducted for approximately 4 months (from 13 Feb 2020 to 8 June 2020).

As shown in Figs. S27(b) and S27(d), the surfaces of the ship were covered by seaweed for 1 month, which shows the requirement of efficient biofouling technology on the commercial ship. The EPC surfaces exhibited relatively higher biofouling properties than the ship surfaces and FAA-free PDMS, indicating the long-term and shear-stable anti-biofouling property of FAAs (Fig. S27(e)). All the OPC surfaces were detached from the surfaces. Surprisingly, the EPC5.0 gel surfaces exhibited almost zero-attachment of marine organisms for 4 months as shown in Figure 5e of the manuscript, demonstrating its sustainable and shear-stable multifunctional lubrication property.

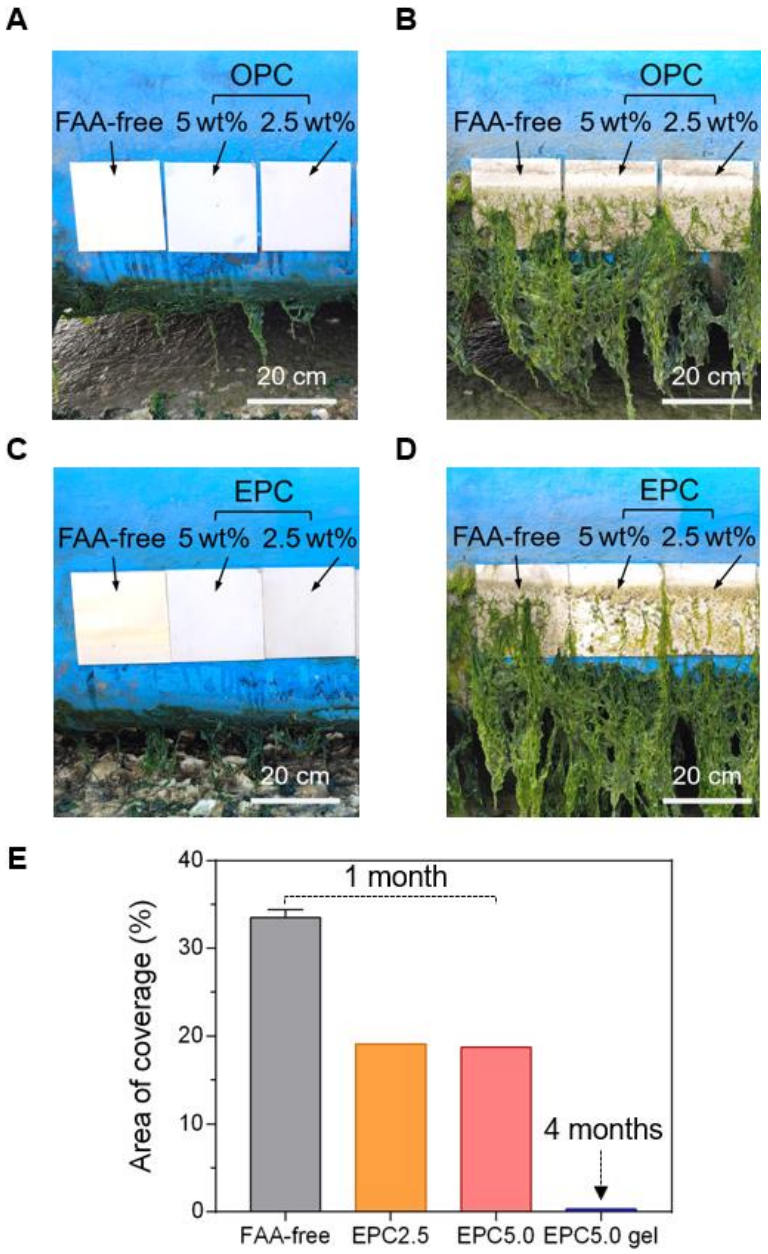

Figure S27. Optical image of FAA-free (PDMS) and OPCs (2.5 and 5.0 wt%) surfaces (a) before the marine field test and (b) after 1 month. Optical image of FAA-free (PDMS) and EPCs (2.5 and 5.0 wt%) surfaces (c) before the marine field test and (d) after 1 month. (e) Quantification of coverage area of bio-foulants on a FAA-free PDMS, EPC2.5, EPC5.0, and EPC5.0 gel.

S3.24. Diverse substrate compatibility of FAA-incorporating oleogels

FAA-incorporating oleogels can be applied to a variety of materials with the aid of primer as follows (Fig. S28(a)): firstly, primer solution was coated on a target substrate. After curing the primer at room temperature, the FAA-incorporating composite solution was coated and cured. The cured composite surface was impregnated with oil. Based on this coating strategy, the FAA-incorporating oleogels were successfully coated on different types of substrates including metal, glass, and plastic, indicating diverse substrate compatibility (Figs. S28(b) and S28(c)).

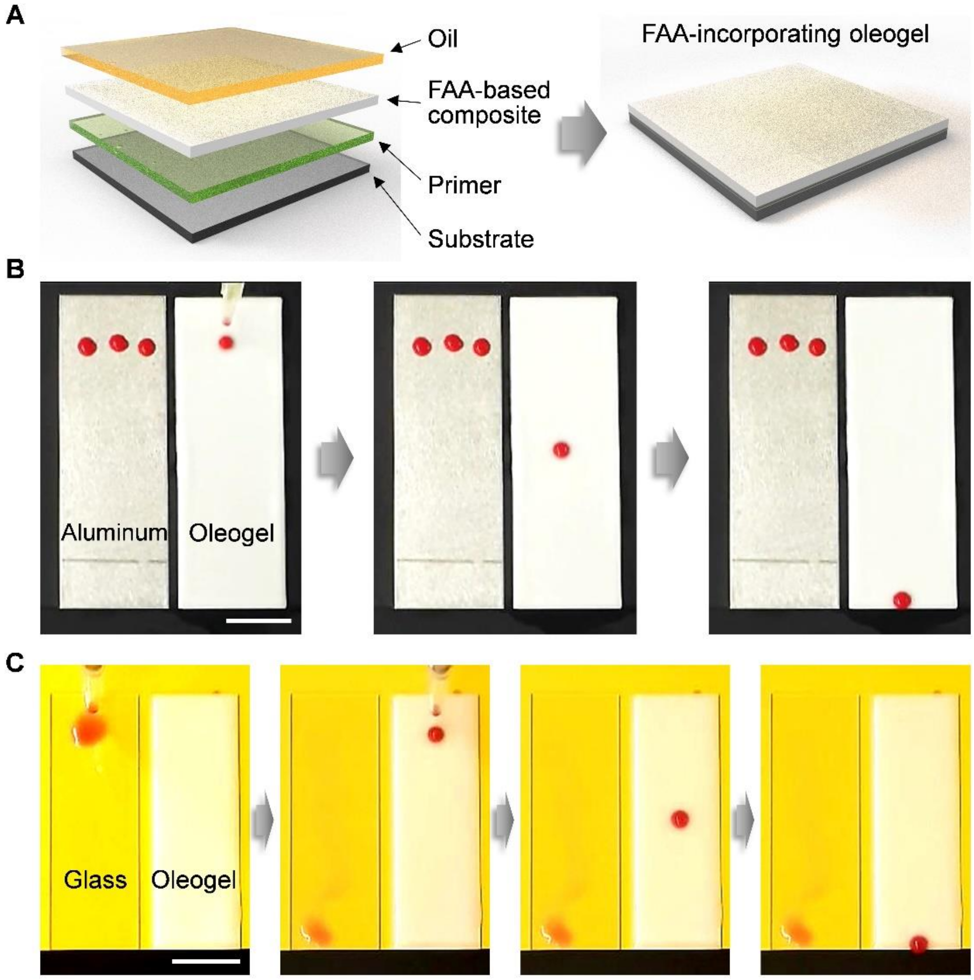

Figure S28. (a) Coating strategy of FAA-incorporating oleogels on various substrates. Sliding motion of water droplets on a FAA-incorporating oleogel-coated (b) aluminium and (c) glass substrate. Water droplets were dyed with a water-soluble red paint for clear visibility

of the droplet motion. The scale bars represent 2 cm.

S3.25. Scalability of FAA-incorporating oleogels

FAA-incorporating oleogels can be scalable using a low-cost doctor-blade coating process for large-scale fabrication (Fig. S29(a)). The doctor-blade coating is a scalable, simple, low-cost, low temperature, solution-based thin film deposition technique that can be compatible with a roll-to-roll fabrication process of large-area films with high throughput.[14] A large scale FAA-incorporating oleogel was fabricated as follows: firstly, FAA-incorporating composite solution was dropped onto a substrate and swiped linearly by a doctor blade. After curing the spread solution, oil was spontaneously infused into the FAA-incorporating composite film by simply immersing the film into an oil bath. Based on this coating process, a large-area FAA-incorporating oleogel film with a size of 90 × 60 cm^2 was successfully fabricated (Fig. S29(b)).

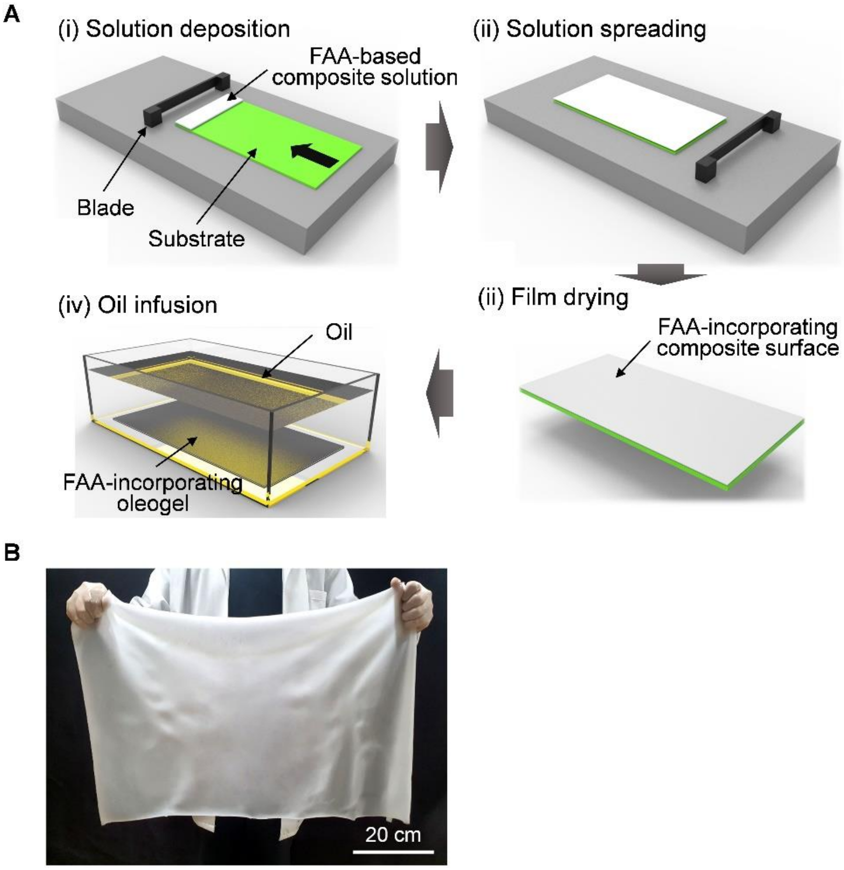

Figure S29. (a) Doctor-blade coating processes of FAA-incorporating oleogels for large scale production. (b) Scaled-up EPC5.0 gel film with a size of 90 × 60 cm^2.

Supplementary References

[1] Ludwig, DB. et al. Flow cytometry: A promising technique for the study of silicone oil-induced particulate formation in protein formulations. *Anal. Biochem.* **410,** 191-199 (2011).

[2] Klochkova, TA. et al. Biology of a terrestrial green alga, Chlorococcum sp. (Chloroc occales, Chlorophyta), collected from the Miruksazi stupa in Korea. *Phycologia*. **45,** 349-358 (2006).

[3] Brochard, F. et al. Dynamical scaling for polymers in theta solvents. *Macromolecules.* **10**, 1157-1161 (1977).

[4] Dangla, R. et al. Microchannel deformations due to solvent-induced PDMS swelling. *Lab Chip*. **10**, 2972-2978 (2010).
[5] Mitchell, GR. et al. The local conformation of Poly(dimethylsiloxane). *Polym. J.* **16**, 351-357 (1984).
[6] Dalod, ARM. et al. Structure and optical properties of titania-PDMS hybrid nanocomposites prepared by in situ non-aqueous synthesis. *Nanomaterials (Basel).* **7**, 460 (2017).
[7] Cai, D. et al. Raman, mid-infrared, near-infrared and ultraviolet–visible spectroscopy of PDMS silicone rubber for characterization of polymer optical waveguide materials. *J. Mol. Struct*. **976**, 274-281 (2010).
[8] Hummel, DO. Atlas of plastics additives. Springer, Berline, Heidelberg (2002).
[9] Delmas, M. et al. Contact Angle Hysteresis at the Nanometer Scale. *Phys. Rev. Lett.* **106**, 136102 (2011).
[10] Kwok, DY. et al. Contact angle measurement and contact angle interpretation. *Adv. Colloid Interface Sci.* **81**, 167-249 (1999).
[11] Brady, RF. et al. Mechanical factors favoring release from fouling release coatings. *Biofouling*. **15**, 73-81 (2000).
[12] DePalma, REBaVA. The relation of the internal surface of grafts to thrombosis, in: Management of Arterial Occlusive Disease (W. A. Dale, ed.). Year book medical publishers (1971).
[13] Griffith, AA. et al. The phenomena of rupture and flow in solids. *Philos. Trans. R. Soc. Lond. Ser. A-Match. Phys. Char*. **221**, 163-198 (1921).
[14] Deng, Y. et al. Scalable fabrication of efficient organolead trihalide perovskite solar cells with doctor-bladed active layers. *Energy Environ. Sci*. **8**, 1544-1550 (2015).
